# M2 macrophage-derived exosomal long non-coding RNA AGAP2-AS1 enhances radiotherapy immunity in lung cancer by reducing microRNA-296 and elevating NOTCH2

**DOI:** 10.1038/s41419-021-03700-0

**Published:** 2021-05-10

**Authors:** Fuquan Zhang, Yonghua Sang, Donglai Chen, Xuejie Wu, Xiaofan Wang, Wentao Yang, Yongbing Chen

**Affiliations:** 1 Department of Cardiothoracic Surgery, The Second Affiliated Hospital of Soochow University, Suzhou, 215004 Jiangsu China; 2 Department of Thoracic Surgery, Shanghai Pulmonary Hospital, Tongji University School of Medicine, Shanghai, 200433 P.R. China

**Keywords:** Cancer, Diseases

## Abstract

Long noncoding RNAs (lncRNAs) and microRNAs (miRNAs) play vital roles in human diseases. We aimed to identify the effect of the lncRNA AGAP2 antisense RNA 1 (AGAP2-AS1)/miR-296/notch homolog protein 2 (NOTCH2) axis on the progression and radioresistance of lung cancer. Expression of AGAP2-AS1, miR-296, and NOTCH2 in lung cancer cells and tissues from radiosensitive and radioresistant patients was determined, and the predictive role of AGAP2-AS1 in the prognosis of patients was identified. THP-1 cells were induced and exosomes were extracted, and the lung cancer cells were respectively treated with silenced AGAP2-AS1, exosomes, and exosomes upregulating AGAP2-AS1 or downregulating miR-296. The cells were radiated under different doses, and the biological processes of cells were assessed. Moreover, the natural killing cell-mediated cytotoxicity on lung cancer cells was determined. The relationships between AGAP2-AS1 and miR-296, and between miR-296 and NOTCH2 were verified. AGAP2-AS1 and NOTCH2 increased while miR-296 decreased in radioresistant patients and lung cancer cells. The malignant behaviors of radioresistant cells were promoted compared with the parent cells. Inhibited AGAP2-AS1, macrophage-derived exosomes, and exosomes overexpressing AGAP2-AS1 or inhibiting miR-296 facilitated the malignant phenotypes of radioresistant lung cancer cells. Furthermore, AGAP2-AS1 negatively regulated miR-296, and NOTCH2 was targeted by miR-296. M2 macrophage-derived exosomal AGAP2-AS1 enhances radiotherapy immunity in lung cancer by reducing miR-296 and elevating NOTCH2. This study may be helpful for the investigation of radiotherapy of lung cancer.

## Introduction

Lung cancer is a major cause of cancer death throughout the world^1^. There are over 1.8 million newly diagnosed lung cancer cases each year and the mortality rate is more than 90%^2^. Lung cancer is traditionally classified into two major histological types, small cell lung cancer (SCLC) (15–25%) and non-SCLC (NSCLC) (75–85%)^3^. The pathogenesis of lung cancer is multifactorial, including environmental and genetic factors, as well as smoking, while the occurrence of lung cancer is related to the regulation of oncogenes and tumor suppressors^4^. As reported, as to 70% of lung cancer patients diagnosed at advanced stages, the 5-year survival rate is roughly 16%. Unfortunately, only 15% of lung cancers were diagnosed at early stages^5^. Radiotherapy exerts a critical effect on SCLC and NSCLC, together with surgery and chemotherapy^6^. Thus, it is important to investigate the molecular mechanism of radiotherapy in lung cancer.

Extracellular vesicles are heterogeneous cell-derived membrane vesicles that released by various cell types, and exosomes are small membrane vesicles with a diameter of 30–150 nm^7^. Macrophages are heterogeneous cells that undergo different functional reprogramming in response to multiple stimulating signals^8^. Human THP-1 macrophages activated by exosomes released by lung adenocarcinoma cells have been revealed to promote lung cancer cell invasion^9^. Long noncoding RNAs (lncRNAs) are transcripts longer than 200 nucleotides but lack significant protein-coding capacity. They regulate multiple biological processes, such as cell growth and differentiation, apoptosis, immune response, and tumorigenesis^10^. It has been reported that lncRNA MALAT-1 protected by exosomes is highly expressed and promotes cell proliferation and migration in NSCLC^11^. AGAP2-AS1 is an antisense lncRNA situated at 12q14.1 and 1567 nt in length that is overexpressed and related to the poor prognosis of NSCLC^12^. Moreover, exosomal AGAP2-AS1 has been confirmed as a diagnostic biomarker of NSCLC^13^. MicroRNAs (miRNAs) are small non-coding RNAs (~22 nt) that bind to the 3′-untranslated region (3′UTR) of target mRNAs and serve as post-transcriptional regulators of mRNA expression^14^. The specific binding region of AGAP2-AS1 and miR-296 was predicted through the RNA22 website. MiR-296 is one of the miRNAs that has been verified to modulate chemo-sensitivity of lung cancer cells^15^, and the role of exosomal miR-296 has been unraveled in hepatocellular carcinoma^16^. Nevertheless, the effect of exosomal miR-296 in lung cancer and the relationship between AGAP2-AS1 and miR-296 have not been explored yet, thus we chose miR-296 as our molecular target for further exploration. RNA22 website also predicted the binding sites between NOTCH2 and miR-296. The Notch signaling pathway is involved in the biological processes of stem cells, progenitor cells, and proliferating cells, and NOTCH2 is one of the Notch family receptors^17^. It has been demonstrated that the balance of the expression of NOTCH1 and NOTCH2 affected the biological behaviors of lung cancer cells^18^. Based on the aforementioned evidence, we could speculate that miR-296 and NOTCH2 might be used as the downstream of AGAP2-AS1 to affect the immune function of lung cancer radiotherapy. This study was performed to identify the role of exosomal AGAP2-AS1 in the progression of lung cancer via the miR-296/NOTCH2 axis, and we might speculate that macrophage-derived exosomal AGAP2-AS1 may influence the immunologic functions of lung cancer patients after radiotherapy with the involvement of miR-296/NOTCH2 axis.

## Results

### AGAP2-AS1 and NOTCH2 expression levels increase while miR-296 expression level decreases in lung cancer cells and tissues

**Fig. 1:**
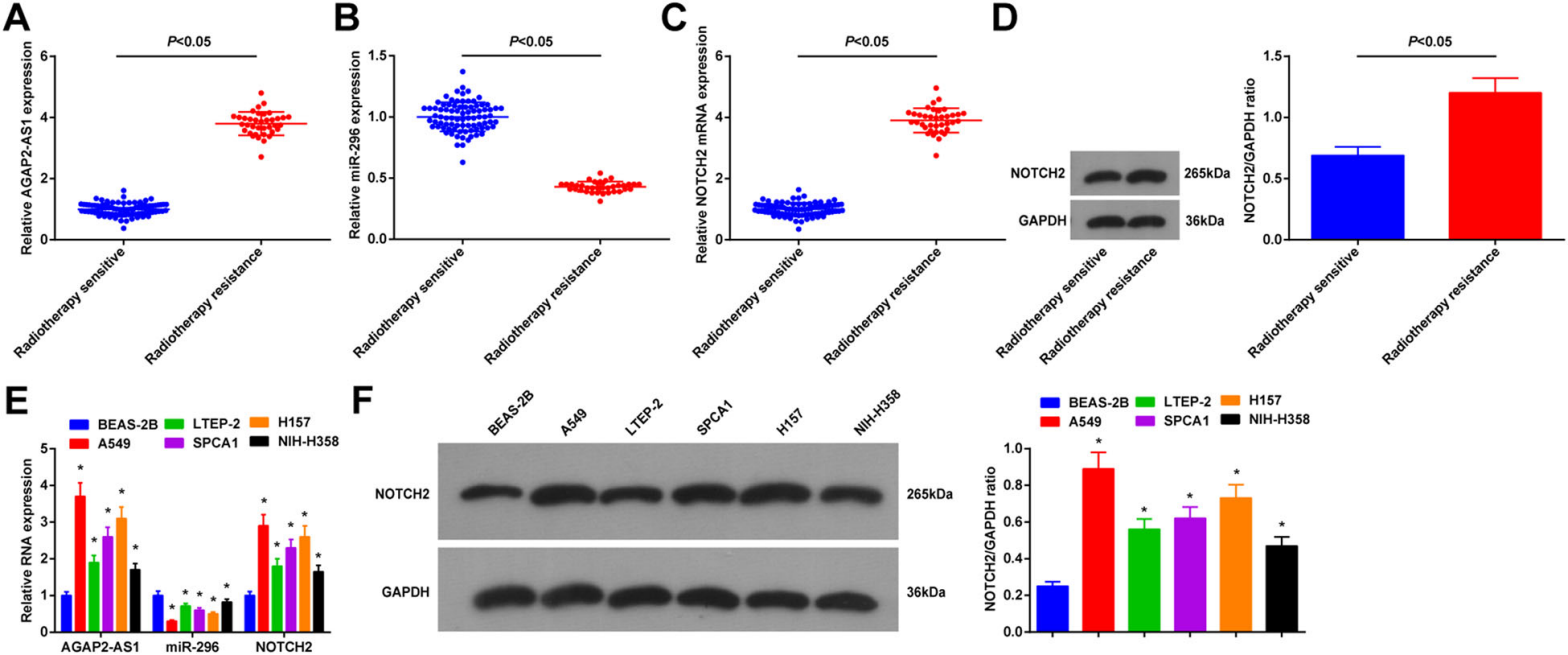
AGAP2-AS1 and NOTCH2 increase while miR-296 decreases in lung cancer cells and tissues. **A** AGAP2-AS1 expression in lung tissues from radiosensitive patients (*n* = 84) and radioresistant patients (*n* = 37). **B** miR-296 expression in lung tissues from radiosensitive patients (*n* = 84) and radioresistant patients (*n* = 37). **C** NOTCH2 expression in lung tissues from radiosensitive patients (*n* = 84) and radioresistant patients (*n* = 37). **D** Protein expression of NOTCH2 in lung tissues from radiosensitive patients (*n* = 84) and radioresistant patients (*n* = 37). **E** Expression of AGAP2-AS1, miR-296, and NOTCH2 in lung cancer cell lines (*N* = 3). **F** Protein expression of NOTCH2 in normal lung epithelial cell BEAS-2B and lung cancer cell lines (*N* = 3); *P < 0.05 vs. BEAS-2B cells; the measurement data were expressed as mean ± standard deviation, unpaired *t* test was performed for comparisons between two groups, one-way ANOVA was used for comparisons among multiple groups and Tukey’s post hoc test was used for pairwise comparisons after one-way ANOVA.

First, AGAP2-AS1, miR-296, and NOTCH2 levels in lung cancer tissues and adjacent tissues were determined by reverse transcription-quantitative polymerase chain reaction (RT-qPCR). Compared with adjacent tissues, AGAP2-AS1 and NOTCH2 expression were elevated while miR-296 expression was reduced in cancer tissues (Supplementary Fig. 1A–C). To identify the roles of AGAP2-AS1, miR-296, and NOTCH2 in radioresistance in lung cancer, we divided lung cancer patients into the radioresistant group and radiosensitive group. Then, AGAP2-AS1, miR-296, and NOTCH2 expression were tested in the two groups. It was found that (Fig. 1A–D) compared with the radiosensitive patients, radioresistant patients had higher levels of AGAP2-AS1 and NOTCH2, as well as a lower level of miR-296. In contrast to BEAS-2B cells, AGAP2-AS1 and NOTCH2 levels increased while miR-296 level decreased in A549, LTEP-2, SPCA1, H157, and NIH-H358 cells (Fig. 1E, F). A549 and H157 cells had larger differences in expression from BEAS-2B cells.

### AGAP2-AS1 plays a predictive role in the prognosis of lung cancer patients

According to the average value of AGAP2-AS1, miR-296, and NOTCH2 expression, the lung cancer patients were separated into the high expression and low expression groups, and the relation between AGAP2-AS1, miR-296, and NOTCH2 expression and clinicopathological characteristics and prognosis of lung cancer patients was analyzed. The results indicated that AGAP2-AS1, miR-296, and NOTCH2 expression levels were related to the tumor, node, and metastasis (TNM) stage and lymph node metastasis (LNM) of lung cancer patients. AGAP2-AS1 expression did not correspond with age, gender, tumor differentiation, and pathological pattern of lung cancer patients (Supplementary Table 2). Kaplan–Meier method was conducted to analyze the relationships of AGAP2-AS1, miR-296, and NOTCH2 levels with the OS and DFS of lung cancer patients. The results showed that patients with AGAP2-AS1 or NOTCH2 high expression had lower OS and DFS. Moreover, patients with high miR-296 expression had high OS and DFS (Supplementary Fig. 2A–C), suggesting that AGAP2-AS1, miR-296, and NOTCH2 are associated with the prognosis of lung cancer patients.

### AGAP2-AS1 negatively regulates miR-296 and NOTCH2 is a target gene of miR-296

An online analysis website (http://lncatlas.crg.eu/) was used to investigate the functional mechanism of AGAP2-AS1, and it was found that (Supplementary Fig. 3A) AGAP2-AS1 mainly distributed in the cytoplasm. This result was confirmed by RNA-FISH assay, and it came out that (supplementary Fig. 3B) AGAP2-AS1 did mainly distribute in the cytoplasm, showing that AGAP2-AS1 may function in cytoplasm.

It was predicted that (Supplementary Fig. 3C, D) there was a specific binding region between the AGAP2-AS1 gene sequence and the miR-296 sequence. It was further confirmed that (Supplementary Fig. 3E) relative to the mimic NC group, the luciferase activity of cells with the AGAP2-AS1-WT in the miR-296 mimic group was repressed. Results of RNA pull-down assay (Supplementary Fig. 3F) reflected that in relation to the Bio-NC group, the enrichment of AGAP2-AS1 was enhanced in the Bio-miR-296-WT group, further indicating that miR-296 particularly bound to AGAP2-AS1. RIP assay was conducted to explore the regulatory role of miRNA in Ago2 protein. The outcomes implied that (Supplementary Fig. 3G) the expression of both AGAP2-AS1 and miR-296 elevated after bound with Ago2, suggesting an interaction between AGAP2-AS1 and miR-296. It was found in dual luciferase report gene assay that (Supplementary Fig. 3H) contrasted to the mimic NC group, the luciferase activity of cells with the NOTCH2-WT in the miR-296 mimic group was suppressed, showing that miR-296 specifically bound to NOTCH2.

### Radioresistant lung cancer cells have stronger radioresistance ability than parental cells

**Fig. 2:**
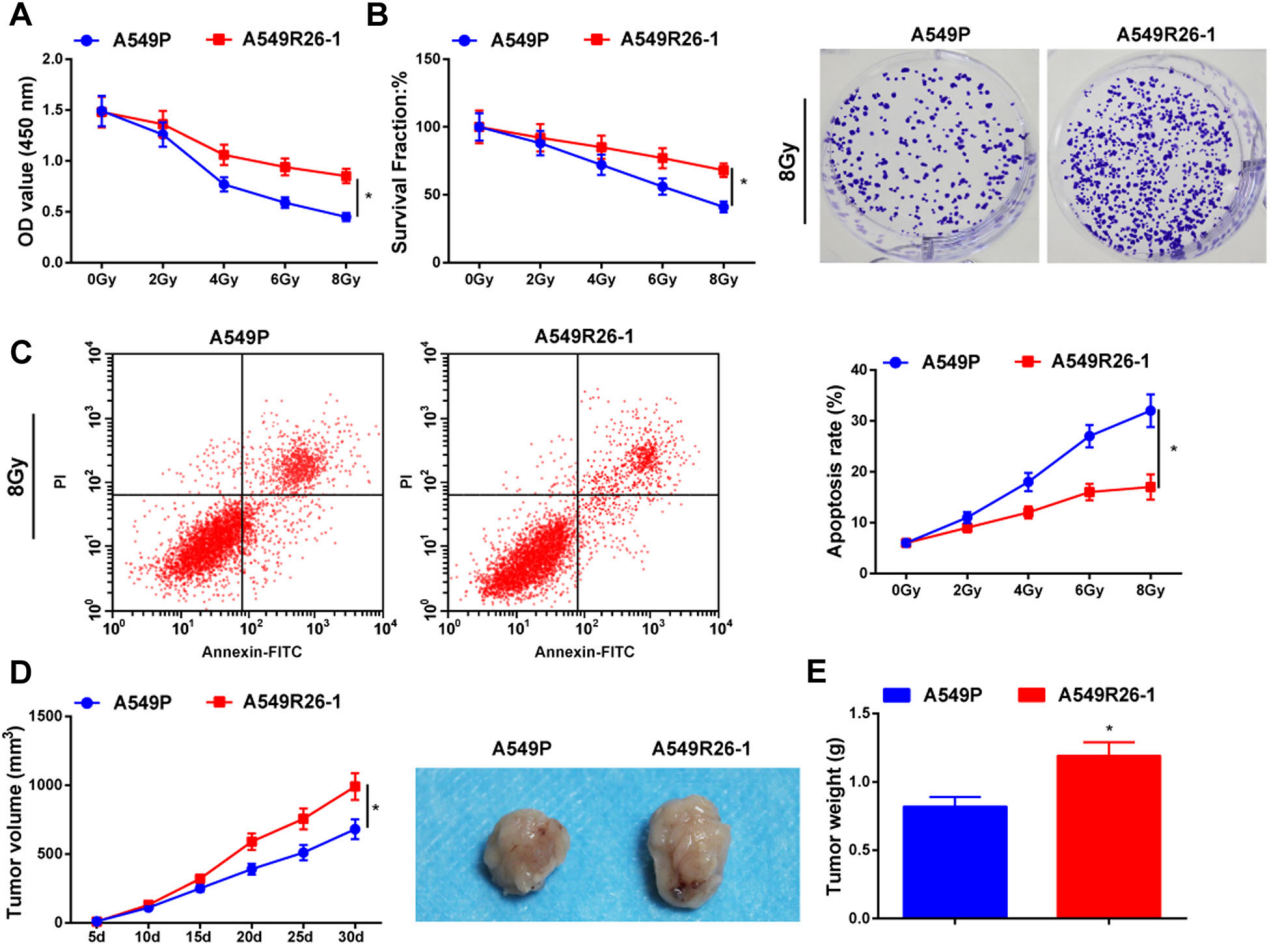
Radioresistant lung cancer cells have stronger radioresistance ability than parent cells. **A** Viability of radioresistant lung cancer cells A549R26-1 and parental cells A549P was determined by CCK-8 assay. **B** Colony formation ability of radioresistant lung cancer cells A549R26-1 and parental cells A549P was detected by colony formation assay. **C** Apoptosis of radioresistant lung cancer cells A549R26-1 and parent cells A549P was detected by flow cytometry. **D** Volume of xenografts from nude mice that had been injected with radioresistant lung cancer cells A549R26-1 and parental cells A549P. **E** Weight of xenografts from nude mice that had been injected with radioresistant lung cancer cells A549R26-1 and parental cells A549P; *N* = 3 in the cellular experiment and *n* = 6 in the animal experiment; *P < 0.05 vs. A549P cells; the measurement data were expressed as mean ± standard deviation, one-way ANOVA was used for comparisons among multiple groups and Tukey’s post hoc test was used for pairwise comparisons after one-way ANOVA.

As to the radiosensitivity of radioresistant lung cancer cells A549R26-1 and H157R24-1, and parental cells A549P and H157P, we performed CCK-8 assay and colony formation assay to evaluate the proliferation and survival rate of cells after radiation. It was observed that (Fig. 2A, B; Supplementary Fig. 4A, B) with the radiation dose increased, the viability and survival rate of cells reduced; treated with the same radiation dose, the proliferation and survival rate of A549R26-1 and H157R24-1 cells were promoted in relation to A549P and H157P cells. Flow cytometry was used to determine the apoptosis of cell lines. We found that (Fig. 2C; Supplementary Fig. 4C) an increased radiation dose resulted in a higher apoptosis rate; exposed to the same radiation dose, the apoptosis rate of A549R26-1 and H157R24-1 cells was suppressed relative to A549P and H157P cells. These data indicated that radioresistant lung cancer cells had stronger radioresistance ability than parental cells.

It was observed in subcutaneous tumorigenesis in nude mice that (Fig. 2D, E; Supplementary Fig. 4D, E) volume and weight of xenografts from nude mice that had been injected with A549R26-1 and H157R24-1 cells were increased compared with nude mice that had been injected with A549P and H157P cells.

### Inhibited AGAP2-AS1 restricts radioresistance of radioresistant lung cancer cells

**Fig. 3:**
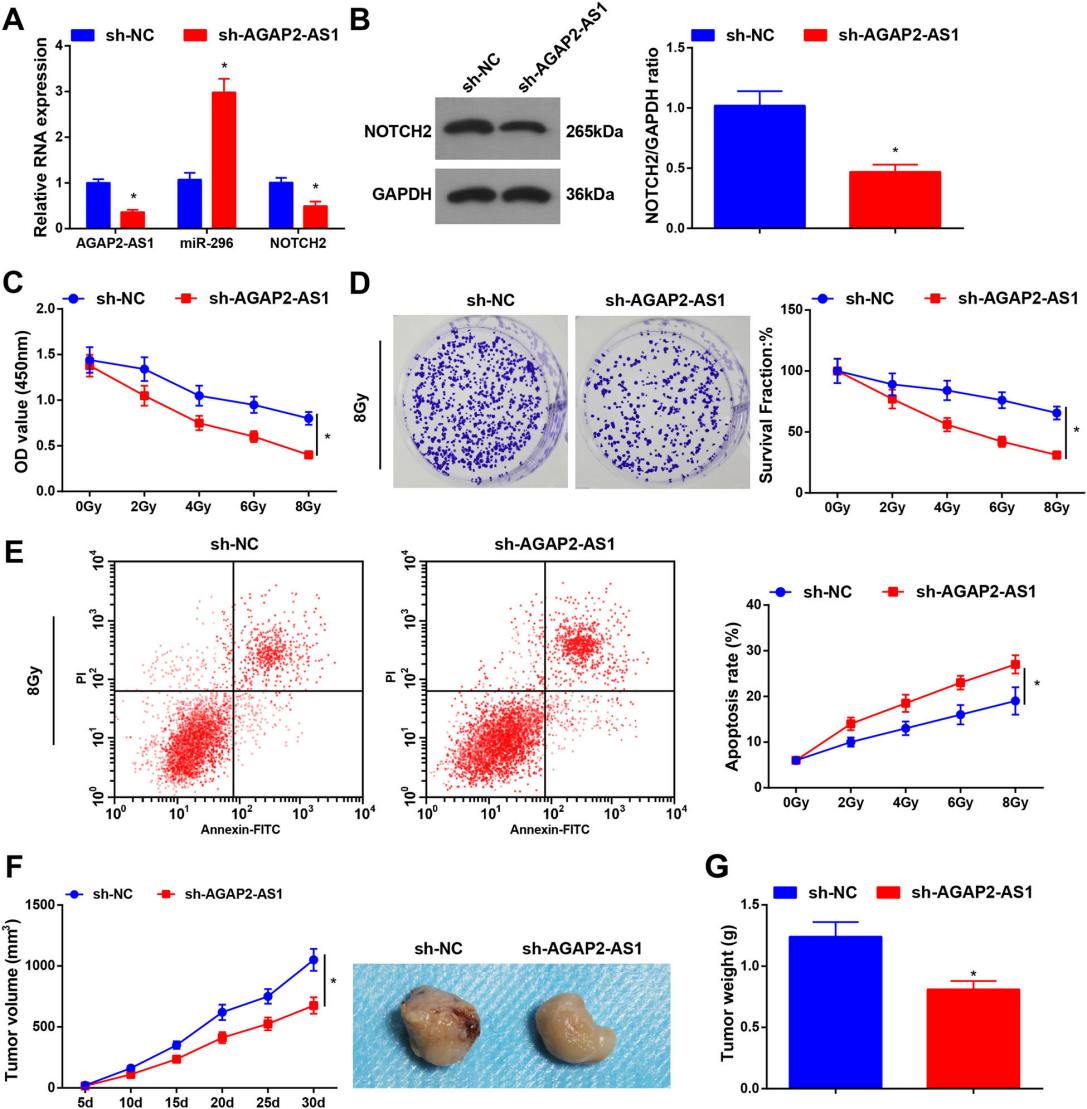
Inhibited AGAP2-AS1 restricts radioresistance of radioresistant lung cancer cells. **A** Expression of AGAP2-AS1, miR-296, and NOTCH2 in A549R26-1 cells was detected by RT-qPCR. **B** Protein expression of NOTCH2 in A549R26-1 cells was determined by Western blot analysis. **C** Viability of radioresistant lung cancer cells A549R26-1 was measured using CCK-8 assay. **D** Colony formation ability of radioresistant lung cancer cells A549R26-1 was evaluated by colony formation assay. **E** Apoptosis of radioresistant lung cancer cells A549R26-1 was determined by flow cytometry. **F** Volume of xenografts from nude mice that had been injected with radioresistant lung cancer cells A549R26-1; **G** weight of xenografts from nude mice that had been injected with radioresistant lung cancer cells A549R26-1; *N* = 3 in the cellular experiment and *n* = 6 in the animal experiment; *P < 0.05 vs. the sh-NC group; the measurement data were expressed as mean ± standard deviation, one-way ANOVA was used for comparisons among multiple groups and Tukey’s post hoc test was used for pairwise comparisons after one-way ANOVA.

AGAP2-AS1, miR-296, and NOTCH2 expression levels were assessed and we found that (Fig. 3A, B; Supplementary Fig. 5A, B) vs. the sh-NC group, AGAP2-AS1, and NOTCH2 expression decreased while miR-296 expression increased in the sh-AGAP2-AS1 group, showing that AGAP2-AS1 was successfully knocked down in A549R26-1 and H157R24-1 cells.

CCK-8 and colony formation assays revealed that (Fig. 3C, D; Supplementary Fig. 5C, D) the viability and survival rate of cells was restricted with the increase of radiation dose; under the same dose of radiation, the viability and survival rate of cells in the sh-AGAP2-AS1 group were repressed relative to the sh-NC group. Flow cytometry results indicated that (Fig. 3E; Supplementary Fig. 5E) with the radiation dose increased, the apoptosis rate of cells was heightened; under the same dose of radiation, the apoptosis rate in the sh-AGAP2-AS1 group was higher than that in the sh-NC group. These data indicated that the downregulation of AGAP2-AS1 constrained the radioresistance of radioresistant lung cancer cells.

Outcomes of subcutaneous tumorigenesis in nude mice suggested that (Fig. 3F, G; Supplementary Fig. 5F, G) the tumor volume and weight of xenografts in the sh-AGAP2-AS1 group were suppressed in relation to the sh-NC group.

### Identification of macrophages and macrophage-derived exosomes

**Fig. 4:**
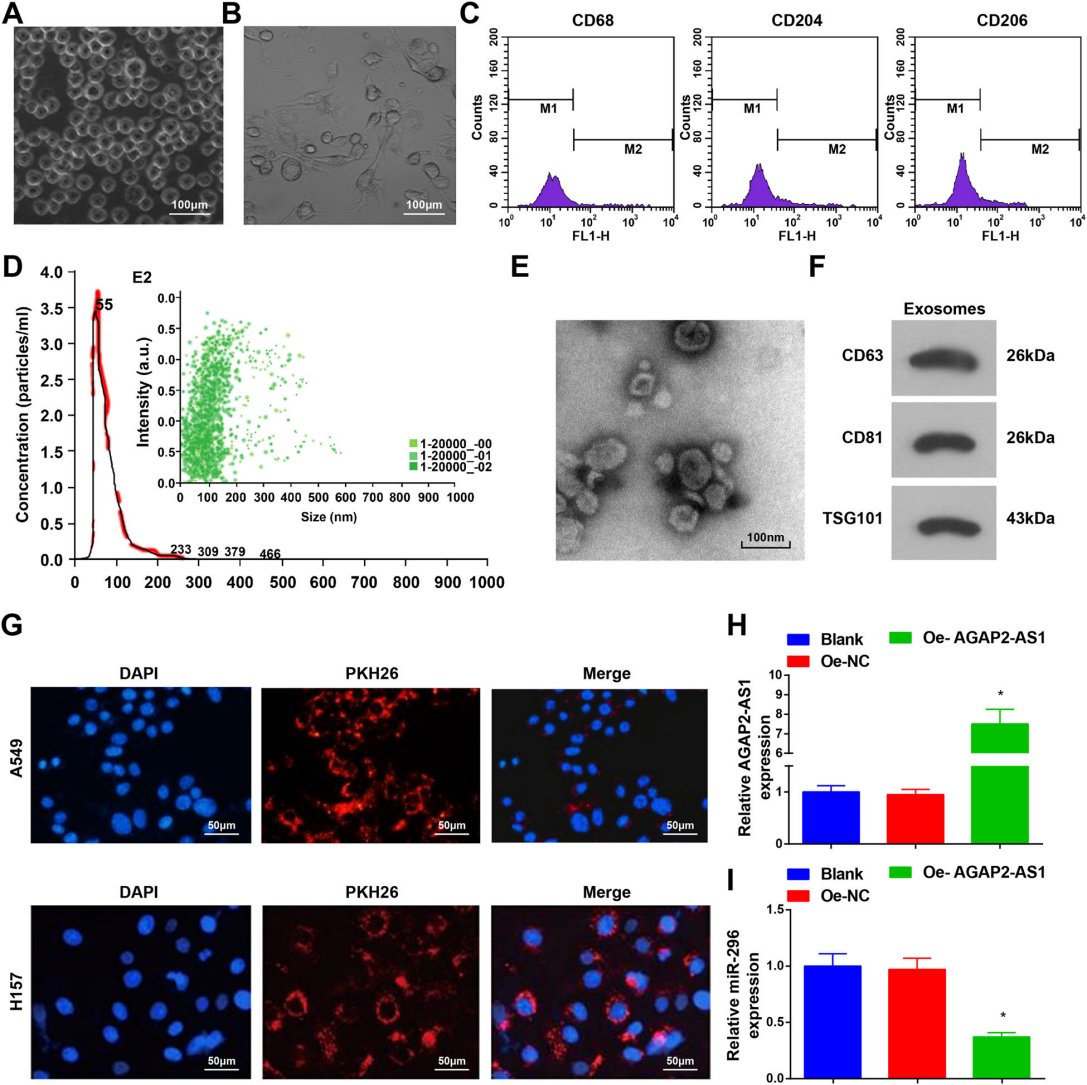
Identification of macrophages and macrophage-derived exosomes. **A** Morphological observation of THP-1 cells. **B** Morphological observation of PMA-induced macrophages. **C** Macrophages were identified by flow cytometry. **D** diameter distribution and particle concentration of exosomes were observed by Nanosight. **E** Exosome morphology was observed under a TEM. **F** Expression of CD63, CD81, and TSG101 was measured by Western blot analysis. **G** Uptake of exosomes. **H** AGAP2-AS1 expression in macrophages. **I** miR-296 expression in macrophages; *N* = 3, *P < 0.05 vs. the Oe-NC group; the measurement data were expressed as mean ± standard deviation, one-way ANOVA was used for comparisons among multiple groups and Tukey’s post hoc test was used for pairwise comparisons after one-way ANOVA.

It was observed through a light microscope that the THP-1 cells were round suspended cells with high viability (Fig. 4A). THP-1 cells became adherent after induction, and then the cells were aggregated, extended pseudopod, and differentiated into adherent cells with irregular morphology (Fig. 4B).

All of the macrophages expressed CD68, CD204, and CD206, so we used a flow cytometer to determine CD68, CD204, and CD206 on the surface of the induced adherent cells. It was found that (Fig. 4C) the markers positively expressed and the expression rate of CD68, CD204, and CD206 was 84.73%, 69.82%, and 65.16%, respectively, which were in line with the characteristic phenotypes of macrophages^19^.

Exosomes were extracted and then identified using NTA, TEM observation, and Western blot analysis. The diameter distribution and particle concentration were observed by Nasosight, and the results suggested that (Fig. 4D) the particle diameter conformed to the singlet normal distribution curve, the peak value was at ~55 nm and the particles distributed between 30 and 180 nm. The particles evenly dispersed in samples. It was observed by a TEM that (Fig. 4E) there were round membranous vesicles with complete envelope, cyathiform structure, and low-density substance. We found in Western blot analysis that (Fig. 4F) all of the exosome surface markers CD63, CD81, and TSG101 were positively expressed.

To measure whether macrophage-derived exosomes maintained the activity when entering cells, the PKH26-labeled exosomes were co-cultured with A549R26-1 and H157R24-1 cells, and DAPI staining reflected that the exosomes had the activity entering A549R26-1 and H157R24-1. Red fluorescence particles indicated the PKH26-labeled exosomes (Fig. 4G).

AGAP2-AS1 and miR-296 expression in macrophages was assessed and we found that (Fig. 4H, I) AGAP2-AS1 overexpressed vector elevated AGAP2-AS1 expression and miR-296 inhibitor reduced miR-296 expression.

### Macrophage-derived exosomes strengthen radioresistance of radioresistant lung cancer cells

**Fig. 5:**
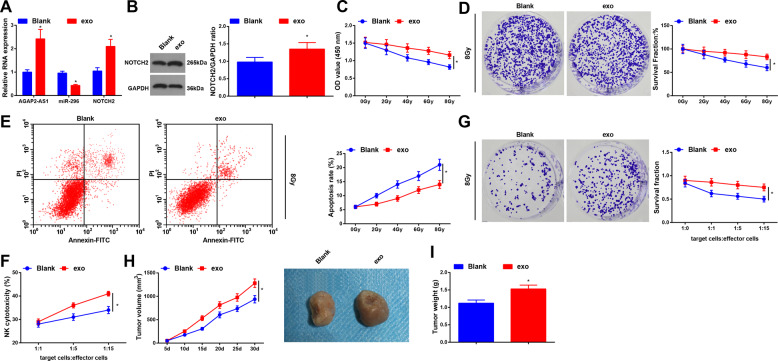
Macrophage-derived exosomes strengthen radioresistance of radioresistant lung cancer cells. **A** Expression of AGAP2-AS1, miR-296, and NOTCH2 in A549R26-1 cells was detected by RT-qPCR. **B** Protein expression of NOTCH2 in A549R26-1 cells was determined by Western blot analysis. **C** Viability of radioresistant lung cancer cells A549R26-1 was measured using CCK-8 assay. **D** Colony formation ability of radioresistant lung cancer cells A549R26-1 was evaluated by colony formation assay. **E** Apoptosis of radioresistant lung cancer cells A549R26-1 was determined by flow cytometry. **F** NK cells-mediated cytotoxicity against radioresistant lung cancer cells A549R26-1 was detected by LDH cytotoxic assay. **G** NK cells-mediated cytotoxicity against radioresistant lung cancer cells A549R26-1 was detected by colony formation assay. **H** Volume of xenografts from nude mice that had been injected with radioresistant lung cancer cells A549R26-1; **I**, weight of xenografts from nude mice that had been injected with radioresistant lung cancer cells A549R26-1; *N* = 3 in the cellular experiment and *n* = 6 in the animal experiment; *P < 0.05 vs. the blank group in A549R26-1 cells; the measurement data were expressed as mean ± standard deviation, one-way ANOVA was used for comparisons among multiple groups and Tukey’s post hoc test was used for pairwise comparisons after one-way ANOVA.

AGAP2-AS1, miR-296, and NOTCH2 expression levels in cells after coculture with exosomes were determined and results implied that (Fig. 5A, B; Supplementary Fig. 6A, B) vs. the blank group, AGAP2-AS1 and NOTCH2 increased while miR-296 decreased in the exo group, suggesting that exosomes transferred AGAP2-AS1 to A549R26-1 and H157R24-1 cells.

CCK-8 and colony formation assays revealed that (Fig. 5C, D; Supplementary Fig. 6C, D) the viability and survival rate of cells was restricted with the increase of radiation dose; under the same dose of radiation, the viability and survival rate of cells in the exo group were promoted relative to the blank group. Results of flow cytometry indicated that (Fig. 5E; Supplementary Fig. 6E) with the radiation dose increased, the apoptosis rate of cells was heightened; under the same dose of radiation, the apoptosis rate in the exo group was lower than that in the blank group. These data suggested that macrophage-derived exosomes promoted the radioresistance of radioresistant lung cancer cells.

Primary NK cells were purified from PBMCs and flow cytometry evaluated that (Supplementary Fig. 1D) the purity of NK cells was (90.6 ± 6.2)%, suggesting that these cells were in accordance with the characteristic phenotype of NK cells^20^.

We found from LDH cytotoxic and colony formation assays that (Fig. 5F; Supplementary Fig. 6F) vs. the blank group, the resistance of A549R26-1 and H157R24-1 cells to NK cell-mediated cytotoxicity was enhanced in the exo group. In colony formation assay (Fig. 5G; Supplementary Fig. 6G), compared with the blank group, the number of colonies in the exo group after cocultured with NK cells was augmented, implying that macrophage-derived exosomes could increase the radioresistance of radioresistant lung cancer cells to NK cytotoxicity.

Outcomes of subcutaneous tumorigenesis in nude mice suggested that (Fig. 5H, I; Supplementary Fig. 6H, I) the tumor volume and weight of xenografts in the exo group were elevated in relation to the blank group.

### Macrophage-derived exosomes elevate AGAP2-AS1 or reduce miR-296 to strengthen radioresistance of radioresistant lung cancer cells

**Fig. 6:**
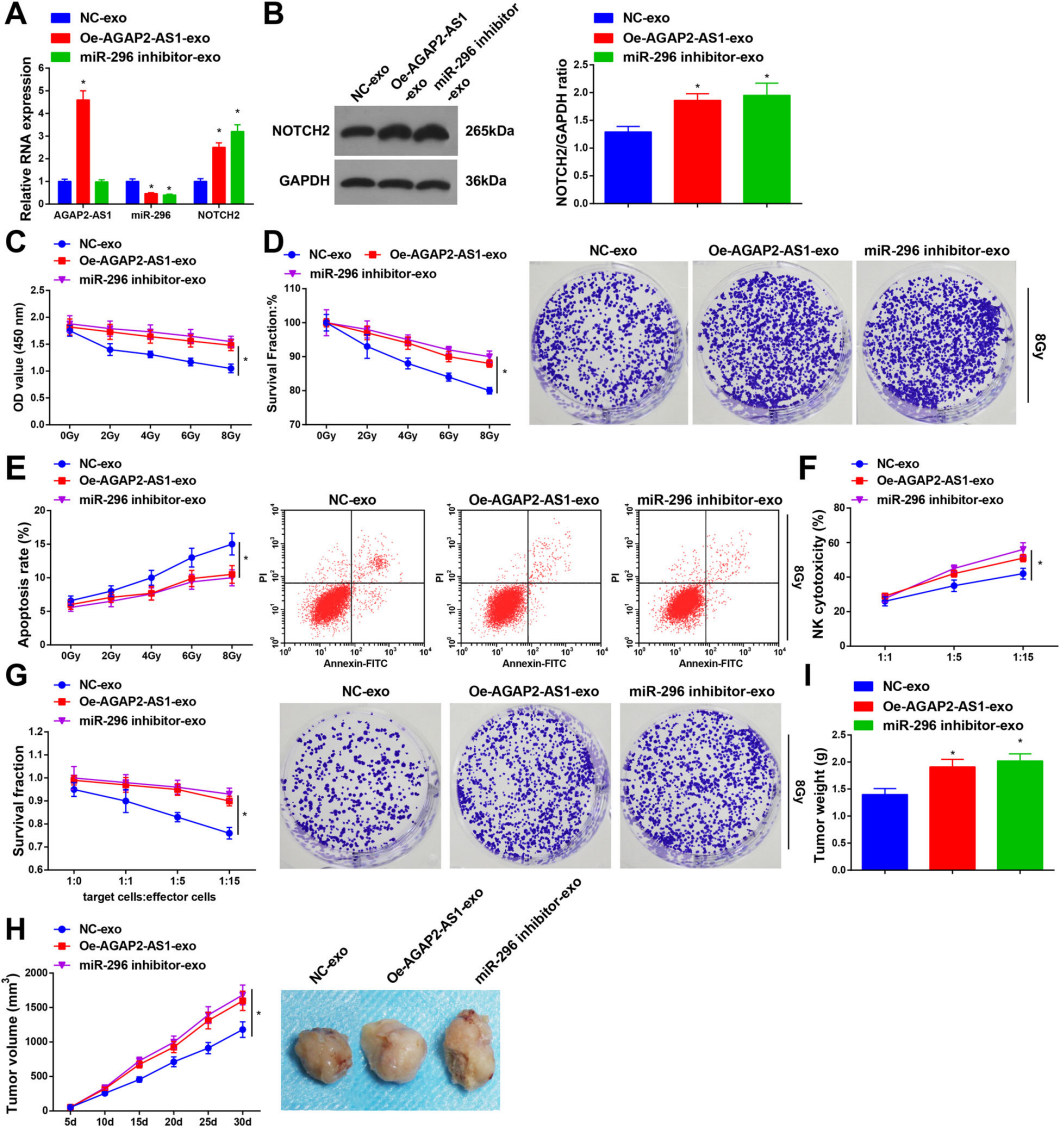
Macrophage-derived exosomes overexpress AGAP2-AS1 or reduce miR-296 to strengthen radioresistance of radioresistant lung cancer cells. **A** Expression of AGAP2-AS1, miR-296, and NOTCH2 in A549R26-1 cells was detected by RT-qPCR. **B** Protein expression of NOTCH2 in A549R26-1 cells was determined by Western blot analysis. **C** Viability of radioresistant lung cancer cells A549R26-1 was measured using CCK-8 assay. **D** Colony formation ability of radioresistant lung cancer cells A549R26-1 was evaluated by colony formation assay. **E** Apoptosis of radioresistant lung cancer cells A549R26-1 was determined by flow cytometry. **F** NK cells-mediated cytotoxicity against radioresistant lung cancer cells A549R26-1 was detected by LDH cytotoxic assay. **G** NK cells-mediated cytotoxicity against radioresistant lung cancer cells A549R26-1 was detected by colony formation assay. **H** Volume of xenografts from nude mice that had been injected with radioresistant lung cancer cells A549R26-1. **I** Weight of xenografts from nude mice that had been injected with radioresistant lung cancer cells A549R26-1; *N* = 3 in the cellular experiment and *n* = 6 in the animal experiment; *P < 0.05 vs. the NC-exo group; the measurement data were expressed as mean ± standard deviation, one-way ANOVA was used for comparisons among multiple groups and Tukey’s post hoc test was used for pairwise comparisons after one-way ANOVA.

Expression of AGAP2-AS1, miR-296, and NOTCH2 was assessed and results implied that (Fig. 6A, B; Supplementary Fig. 7A, B) vs. the NC-Exo group, AGAP2-AS1 and NOTCH2 increased while miR-296 decreased in the Oe-AGAP2-AS1-Exo group, and NOTCH2 elevated while miR-296 reduced in the miR-296 inhibitor-exo group, implying that exosomes upregulated AGAP2-AS1 or downregulated miR-296, and transferred them to A549R26-1 and H157R24-1 cells.

To explore the role of macrophage-derived exosomes overexpressing AGAP2-AS1 or inhibiting miR-296 in radiosensitivity of A549R26-1 and H157R24-1 cells, CCK-8, and colony formation assays were performed. It was found that (Fig. 6C, D; Supplementary Fig. 7C, D) the viability and survival rate of cells were restricted with the increase of radiation dose; under the same dose of radiation, the viability and survival rate of cells in the Oe-AGAP2-AS1-exo group and the miR-296 inhibitor-exo group were promoted relative to the NC-exo group. Results of flow cytometry indicated that (Fig. 6E; Supplementary Fig. 7E) with the radiation dose increased, the apoptosis rate of cells was heightened; under the same dose of radiation, the apoptosis rate in the Oe-AGAP2-AS1-exo group and the miR-296 inhibitor-exo group was lower than that in the NC-exo group. These data indicated that macrophage-derived exosomes overexpressing AGAP2-AS1 or reducing miR-296 promoted the radioresistance of radioresistant lung cancer cells.

LDH cytotoxic and colony formation assays evaluated the role of macrophage-derived exosomes elevating AGAP2-AS1 or repressing miR-296 in resistance of A549R26-1 and H157R24-1 cells to NK cell-mediated cytotoxicity. We discovered that (Fig. 6F; Supplementary Fig. 7F) in comparison to the NC-exo group, the resistance of A549R26-1 and H157R24-1 cells to NK cell-mediated cytotoxicity was enhanced in the Oe-AGAP2-AS1-exo group and the miR-296 inhibitor-exo group. In colony formation assay (Fig. 6G; Supplementary Fig. 7G), compared with the NC-exo group, the number of colonies in the Oe-AGAP2-AS1-exo group and the miR-296 inhibitor-exo group after cocultured with NK cells was increased, respectively. These findings indicated that macrophage-derived exosomes overexpressing AGAP2-AS1 or inhibiting miR-296 enhanced the resistance of A549R26-1 and H157R24-1 cells to NK cell-mediated cytotoxicity.

Outcomes of subcutaneous tumorigenesis in nude mice suggested that (Fig. 6H, I; Supplementary Fig. 7H, I) the tumor volume and weight of xenografts in the Oe-AGAP2-AS1-exo group and the miR-296 inhibitor-exo group were elevated in relation to the NC-exo group.

### NOTCH2 overexpression rescues the effects of miR-296 upregulation on radioresistance of lung cancer cells

**Fig. 7:**
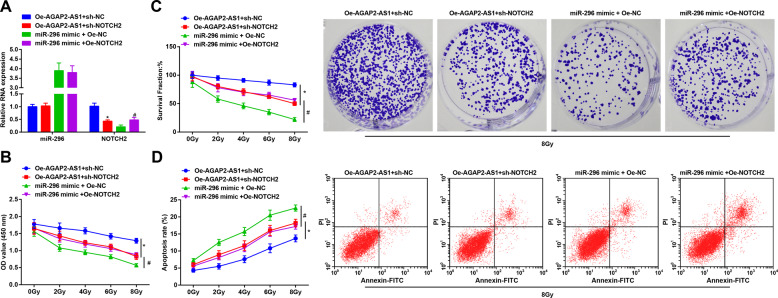
NOTCH2 overexpression rescues the effects of miR-296 upregulation on radioresistance of radioresistant lung cancer cells. **A** Detection of transfection efficiency of A549R26-1 cells. **B** Proliferation of A549R26-1 cells was detected by CCK-8 assay. **C** Survival rate of A549R26-1 cells was detected by colony formation assay. **D** Apoptosis rate of A549R26-1 cells was detected by flow cytometry. *N* = 3; *P < 0.05 vs. the Oe-AGAP2 + sh-NC group, ^#^*P* < 0.05 vs. the miR-296 mimic + Oe-NC group; the measurement data were expressed as mean ± standard deviation, one-way ANOVA was usedfor comparisons among multiple groups and Tukey’s post hoc test was used for pairwise comparisons after one-way ANOVA.

To further explore whether AGAP2-AS1 and miR-296 function through modulating NOTCH2, rescue experiments were performed win A549R26-1 and H157R24-1 cells. RT-qPCR was performed to assess miR-296 and NOTCH2 expression to confirm the transfection efficiency (Fig. 7A; Supplementary Fig. 8A).

CCK-8 assay, colony formation assay and flow cytometry revealed that cell viability and cell survival rate were diminished while cell apoptosis was raised in a dosed manner. Treated with the same irradiation dose, versus the Oe-AGAP2-AS1 + sh-NC group, cell viability, and cell survival rate were impaired while cell apoptosis was promoted in the Oe-AGAP2-AS1 + sh-NOTCH2 group. By comparison with the miR-296 mimic + Oe-NC group, enhanced cell viability and cell survival rate, as well as inhibited cell apoptosis was found in the miR-296 mimic + Oe-NOTCH2 group (Fig. 7B–D; Supplementary Fig. 8B–D). It was implied that NOTCH2 could be used as a downstream target of AGAP2-AS1 and miR-296 in radioresistant lung cancer cells.

## Discussion

Due to the side effects of radiotherapy in lung cancer patients, it is urgent to explore factors related to tumor radiation resistance, thereby developing radiosensitizers that increase the efficacy of radiotherapy and suppress the required radiation dose through targeting these factors^21^. Exosomes are critical mediators of intercellular communication that have been confirmed to transfer lipids, proteins, mRNAs, and miRNAs to recipient cells^22^. We conducted this research to identify the roles of M2 macrophage-derived exosomal AGAP2-AS1 in the development of lung cancer, and we found that exosomal AGAP2-AS1 promoted radiotherapy immunity of lung cancer patients after radiotherapy by downregulating miR-296 and overexpressing NOTCH2.

To begin with, we determined AGAP2-AS1, miR-296, and NOTCH2 expression in lung cancer tissues and cell lines, and the results indicated that AGAP2-AS1 and NOTCH2 increased while miR-296 decreased in radioresistant lung cancer tissues and cells lines, respectively compared with radiosensitive tissues and normal lung epithelial cell BEAS-2B. Consistently, Fan et al.^23^ have found that AGAP2-AS1 is highly expressed in NSCLC tissues vs. the adjacent normal tissues, and recent literature has confirmed that the expression of AGAP2-AS1 is evidently elevated in NSCLC tissues^24^. In addition, miR-296-5p has been found to be downregulated in both NSCLC tissues and cell lines^25^, and Qian et al.^26^ have discovered that the expression of NOTCH2 mRNA is higher in NSCLC tissues than in adjacent non-tumor tissues. Furthermore, we analyzed the relation between AGAP2-AS1 expression and clinicopathological characteristics of lung cancer patients, and it came out that the expression of AGAP2-AS1 was associated with TNM stage and LNM of lung cancer patients, and the higher AGAP2-AS1 expression corresponded with a lower survival rate. Similarly, it has been reported that AGAP2-AS1 expression is related to TNM stage and LNM of NSCLC patients and the high expression of AGAP2-AS1 indicated a poor prognosis^23^. To unravel the effect of macrophage-derived exosomes on the expression of AGAP2-AS1 and miR-296, the lung cancer cells were cocultured with exosomes and we found that the exosomes overexpressed AGAP2-AS1 while reduced miR-296. In line with this result, Tao et al.^13^ have affirmed that AGAP2-AS1 expression in serum exosomes is higher than in exosomes-depleted serum from NSCLC patients, and exosomal miR-296 has been revealed to down-regulate in hepatocellular carcinoma cells^16^. We also confirmed the target relationships between AGAP2-AS1 and miR-296, and between miR-296 and NOTCH2, both of which have been seldom studied.

Outcomes of cellular and animal experiments in our study suggested that macrophage-derived exosomes, exosomal AGAP2-AS1, and downregulated miR-296 promoted malignant behaviors and resistance to NK-cells mediated cytotoxicity of lung cancer cells via regulation of NOTCH2. Consistently, Lobb et al.^27^ have illuminated that exosomes derived from mesenchymal NSCLC cells promote chemoresistance, and it has been recently demonstrated that exosomes transfer MALAT-1 to accelerate proliferation and migration in NSCLC^11^. Moreover, a recent publication has revealed that with an extended treatment time (48 h), the tumor-derived exosomes inhibit the cytotoxicity of NK cells via suppressing activated receptor expression on NK cells^28^. As for the effects of AGAP2-AS1, Li et al.^24^ have unveiled that degradation of AGAP2-AS1 represses NSCLC cell growth, and also constrains tumor growth in vivo. It has also been verified that upregulated exosomal AGAP2-AS1inhibits trastuzumab-induced cytotoxicity in breast cancer cells^29^. In addition, Luo et al.^15^ have unearthed that miR-296-3p restricts NSCLC cell proliferation, and promotes drug resistance and apoptosis, and it has been recently reported that miR-296 restoration restrains malignant behaviors of breast cancer cells and also inhibits in vivo breast cancer cell growth^30^. In fact, activated NOTCH2 signaling enhances chemoresistance to neural stem cells^31^. Mechanistically, decreased NOTCH2 could suppress the cancer stem cell-like properties and chemoresistance in non-small cell lung cancer^32^ and inactivation of NOTCH signaling subdue stemness and chemoresistance in colorectal cancer^33^.

In a word, we clarified that exosomal AGAP2-AS1 promotes the immunologic function of lung cancer patients after radiotherapy via down-regulating miR-296 and upregulating NOTCH2. This research may contribute to further the understanding of molecular mechanisms of radiotherapy in lung cancer.

## Materials and methods

### Ethics statement

Written informed consent was acquired from all patients before this study. The protocol of this study was confirmed by the Ethic Committee of The Second Affiliated Hospital of Soochow University. Animal experiments were strictly in accordance with the Guide to the Management and Use of Laboratory Animals issued by the National Institutes of Health. The protocol of animal experiments was approved by the Institutional Animal Care and Use Committee of The Second Affiliated Hospital of Soochow University.

### Study subjects

A total of 121 lung cancer patients (73 males and 48 females, aged 40–67 years, 64 cases of squamous carcinoma, and 57 cases of adenocarcinoma) that accepted treatment in The Second Affiliated Hospital of Soochow University from May 2010 to December 2015 were selected. Cancer tissues and adjacent tissues were collected from the patients. All of the patients have been pathologically diagnosed and received three-dimensional conformal radiation therapy after the surgery (2 Gy/time, 5 times/w, total dose was 50–70 Gy and the treatment lasted for 8 weeks). Patients were separated into complete remission (CR), partial remission (PR), progressive disease (PD), and stable disease (SD) according to the criterion proposed by World Health Organization. CR and PR were defined as radiosensitive and PD and SD were defined as radioresistant. There were 37 radioresistant cases and 84 radioresistant cases. The follow-up information of the patients was obtained through the telephone and outpatient referral. The survival time of patients was calculated from the time point of diagnosis. The follow-up visit lasted for 3 years and ceased on December 30, 2018. The Kaplan–Meier method was conducted to analyze the correlations of AGAP2-AS1, miR-296, and NOTCH2 expression levels with overall survival (OS) and disease-free survival (DFS) of lung cancer patients. OS referred to the time from randomized grouping to death caused by any reasons. DFS referred to the time from randomized grouping to disease recurrence or death due to disease progression.

### Cell culture

THP-1 monocytes, normal lung epithelial cell BEAS-2B, and human lung cancer cell lines A549, H157, LTEP-2, NIH-H358, and SPCA1 (all obtained from American Type Culture Collection, VA, USA) were incubated with Roswell Park Memorial Institute (RPMI) 1640 medium at 37 °C and with 5% CO_2_.

### Induction and identification of macrophages

Cells in each culture system were incubated with phorbol 12-myristate 13-acetate (PMA, 5 nmol/L) for 48 h, and the THP-1 cells turned into macrophages adhering to the wall. Subsequently, each culture system was added with human recombinant interleukin (IL)-4 and continuously incubated for 24 h. By this time, the cell morphology did not apparently change compared with 24 h before. The macrophages were trypsinized, passaged, and 1 × 10^7^ cells were resuspended using phosphate-buffered saline (PBS) for 3 times with the supernatant discarded. Next, the cells were fixed and centrifuged. Cells were incubated with diluted primary antibodies (all 1:100, anti-CD68, anti-CD204, and anti-CD206, Santa Cruz Biotechnology Inc., CA, USA) at 4 °C for 1 h and centrifuged with the supernatant removed, then incubated with a secondary antibody with light avoidance for 45 min. A flow cytometer (FACSCalibur, BD Biosciences, NJ, USA) was applied for the examination.

### Transfection and grouping of macrophages

P5 macrophages were transfected using lipofectamine^TM^ 2000 (Invitrogen Inc., CA, USA) when cell confluence reached 80%. The sequences were obtained from GenePharma Co., Ltd. (Shanghai, China). Cells were divided into 9 groups: the blank group (untreated macrophages), the overexpressed (Oe)-negative control (NC) group (transfection of AGAP2-AS1 overexpressed vector NC), the Oe-AGAP2-AS1 group (transfection of AGAP2-AS1 overexpressed vector), the inhibitor-NC group (transfection of miR-296 inhibitor NC), the miR-296 inhibitor group (transfection of miR-296 inhibitor), the Oe-AGAP2-AS1 + sh-NC group (transfection of AGAP2-AS1 overexpressed vector and sh-NOTCH2 NC), Oe-AGAP2-AS1 + sh-NOTCH2 group (transfection of AGAP2-AS1 overexpressed vector and sh-NOTCH2), miR-296 mimic + Oe-NC group (transfection of miR-296 mimic and overexpressed NOTCH2 vector NC), and miR-296 mimic + Oe-NOTCH2 group (transfection of miR-296 mimic and overexpressed NOTCH2 vector).

### Extraction and identification of exosomes

Exosomes were extracted from M2 macrophages and purified^34^. M2 macrophages were centrifuged at 500*g* for 15 min, 3000*g* for 15 min, and 12,000*g* for 30 min to remove cells and debris. Exosomes were purified by centrifugation at 140,000*g* for 80 min and resuspended by PBS, then repeatedly purified by ultracentrifugation.

Transmission electron microscopy (TEM) observation: exosome suspension (50 µL) was put on a copper mesh with supporting membranes, then placed for 5 min and dried. The exosome was added with 50 µL 3% phosphotungstic acid solution, stained by 3% phosphotungstic acid dye solution for 5 min, then dried under incandescent light and photographed by a TEM (Hitachi, Ltd., Tokyo, Japan).

Western blot analysis: CD63, tumor susceptibility gene 101 (TSG101), and CD81 (all 1:1000, Abcam, MA, USA) were detected.

Nanoparticle tracking analysis (NTA) was used for the quantitative determination of exosomes: the concentration of exosome samples was diluted into 10^8^–10^9^ particles/mL, and a 40 μL sample was infused into the sample well. The detection threshold was maintained at 5 and the parameters were kept unchanged during the measurement.

### Culture of radioresistant cell lines

Parental A549 and H157 cells (A549P and H157P cells) were radiated with 2–6 Gy each week for 4–5 weeks. Further radiotherapy was performed every week when the surviving cells continued growing. The A549P cells accepted 26 Gy radiation and H157P cells that accepted 24 Gy radiation were named A549P/A549R26-1 and H157P/H157R24-1 cells, respectively.

### Uptake of macrophage-derived exosomes

The cell suspension was seeded onto a laser confocal dish and incubated for 24 h, and the cells adhered to the wall. The fluorochrome PKH26 (Sigma-Aldrich Chemical Company, MO, USA) was employed to stain the exosomes without light exposure for 5 min and the stained exosomes (100 µL) were cocultured with A549R26-1 and H157R24-1 cells for 6 h. The exosomes were observed and photographed under a laser confocal microscope.

### Cell transfection and grouping

A549R26-1 and H157R24-1 cells were separated into several groups: the blank group (no treatment), the short hairpin RNA (sh)-NC group (cells were introduced with NC of AGAP2-AS1 silenced vector), the sh-AGAP2-AS1 group (cells were introduced with AGAP2-AS1 silenced vector), the exo group (cells were co-cultured with untreated macrophage-derived exosomes), the Oe-NC-exo group (cells were co-cultured with exosomes that released from macrophages in the oe-NC group), the Oe-AGAP2-AS1-exo group (cells were cocultured with exosomes that released from macrophages in the Oe-AGAP2-AS1 group), the inhibitor-NC-exo group (cells were cocultured with exosomes that released from macrophages in the inhibitor-NC group), the miR-296 inhibitor-exo group (cells were cocultured with exosomes that released from macrophages in the miR-296 inhibitor group), Oe-AGAP2-AS1 + sh-NC group (cells were cocultured with exosomes that released from macrophages in the Oe-AGAP2-AS1 + sh-NC group), Oe-AGAP2-AS1 + sh-NOTCH2 group (cells were cocultured with exosomes that released from macrophages in the Oe-AGAP2-AS1 + sh-NOTCH2 group), miR-296 mimic + Oe-NC group (cells were cocultured with exosomes that released from macrophages in the miR-296 mimic + Oe-NC group), and miR-296 mimic + Oe-NOTCH2 group (cells were cocultured with exosomes that released from macrophages in the miR-296 mimic + Oe-NOTCH2 group). The exosomes (20 μg) were resuspended using RPMI 1640 medium containing 10% fetal bovine serum (FBS) and cocultured with A549R26-1 or H157R24-1 cells for 48 h.

### Cell counting kit-8 (CCK-8) assay

This assay was conducted as previously described^35^ and a microplate reader (Multiskan FC, Thermo Fisher Scientific Inc., MA, USA) was used for the detection of cell proliferation.

### Colony formation assay

The transfected cells were trypsinized, made into a suspension, and diluted at gradient multiple, then seeded into culture dishes at 1000 cells/dish and incubated for 12 h. Afterward, the cells were exposed to different doses (0, 2, 4, 6, and 8 Gy) of radiation (Cs-137 radiation source, radiation dose rate at 180–205 cGy/min). Cultured for 10 days, the cells were fixed with methanol, stained with 0.5% crystal violet dye solution (w/v), and air-dried. Gridlines were marked and the number of colonies was counted.

### Flow cytometry

Apoptosis was assessed using flow cytometry referring to a publication^36^ and the apoptosis rate was calculated.

### Extraction and measurement of natural killer (NK) cells

NK cell isolation kits (Miltenyi Biotec, Gladbach, Germany) were used to purify primary NK cells from peripheral blood mononuclear cells (PBMCs) of healthy people. The isolated cells were kept in IL-2 containing NK cell media and stained with anti-CD56-PE (e-Bioscience, CA, USA) and anti-CD3-Cy7 (BioLegend, CA, USA). Flow cytometry was employed to measure the purity of separated cells (CD56^+^CD3^−^) and the cells were cultured in α-minimum essential medium containing sodium bicarbonate (Sigma), IL-2 (100 units/mL), inositol (0.2 mM), 2-mercaptoethanol (0.1 mM), folic acid (0.02 mM), 12.5% horse serum and 12.5% FBS (Hyclone Laboratories, Logan, UT).

### Lactic dehydrogenase (LDH) cytotoxic assay

LDH cytotoxic assay was conducted to analyze the killing effect of NK cells. Three thousand lung cancer cells were plated and on the next day, NK cells were supplemented at various ratios. Lung cancer cells (target cells):NK cells (effector cells) = 1:1, 1:5, and 1:15. After 4-h culture, an aliquot of 50 μL medium was used in LDH cytotoxic assay using the LDH cytotoxic assay kit (Thermo Scientific). The experimental release was corrected by subtraction of the spontaneous release of effector cells at corresponding dilutions and the cytotoxicity was calculated.

### Determination of cytotoxicity of NK cells

Three thousand lung cancer cells were seeded onto 10-cm culture dishes and added with NK cells at different ratios (target cells:effector cells = 1:1, 1:5, and 1:15) on the second day. NK cells were removed and fresh medium was appended into lung cancer cells after 4-h co-culture. The colony formation was observed through crystal violet staining after the cells were cultured for 10 days, and the cells were then counted under a microscope.

### Fluorescence in situ hybridization (FISH) assay

According to the directions of FISH kits (Guangzhou RiboBio Co., Ltd., Guangdong, China), A549 and H157 cells were trypsinized, seeded into confocal dishes and cultured, and then the medium was discarded after cell adherence. Fixed with 1 mL formaldehyde for 15 min, the cells were permeabilized with prepared 1% Triton X-100 for 5 min and blocked with prepared prehybridization solution for 30 min, and the hybridization solution was preheated at 37 °C. The prehybridization solution was removed and cells were incubated with probe hybridization solution containing appropriate probes overnight, rinsed by sodium citrate buffer for 3 times (10 min/time), and stained with 4′,6-diamidino-2-phenylindole 2 hci (DAPI) for 10 min. Cells were photographed under a fluorescence microscope.

### Dual-luciferase reporter gene assay

The synthesized AGAP2-AS1 3′UTR gene section was introduced into pMIR-reporter by endonucleases Spe I and Hind III, and the complementary mutant sites of AGAP2-AS1 wild-type (WT) seed sequence were designed. After digestion, T4 DNA ligase was employed to insert the target segment into a pMIR-reporter plasmid. The sequenced luciferase reporter plasmids WT and mutant type (MUT) were co-transfected with miR-296 into A549 and H157 cells for 48 h, and the cells were lysed. Luciferase detection kits (Beyotime Institute of Biotechnology) and a Glomax20/20 luminoscope (Promega, Madison, WI, USA) were utilized to measure the luciferase activity. The relation between miR-296 and NOTCH2 was assessed with the same method.

### RNA pull-down assay

A549 and H157 cells were treated with 50 nM biotin-labeled Bio-miR-296-Wt, Bio-miR-296-Mut, and relative Bio-NC for 48 h. These cells were cultured with a specific lysis buffer solution (Ambion, TX, USA) for 10 min and centrifuged at 14,000*g* with the supernatant collected. The protein lysate was cultured with M-280 streptavidin beads (Sigma) that had been coated with bovine serum albumin at 4 °C for 3 h, then successively washed by pre-cooled pyrolysis buffer (twice), low salt buffer (three times), and high salt buffer (once). Expression of AGAP2-AS1 was determined using reverse transcription-quantitative polymerase chain reaction (RT-qPCR).

### RNA-immunoprecipitation (RIP) assay

RIP assay was performed according to the description in a publication^32^. Trizol was used to extract RNA and RT-qPCR was employed to assess AGAP2-AS1 and miR-296 expression in A549 and H157 cells.

### RT-qPCR

Trizol kits (Invitrogen) were utilized to extract the total RNA in tissues, exosomes and cells, and the RNA was reversely transcribed into cDNA according to the manufacturer’s information (Fermentas Inc., CA, USA). Expression of AGAP2-AS1, miR-296, and NOTCH2 was assessed using SYBR Premix Ex Taq II Kits (Takara Bio Inc., Shiga, Japan). U6 was used as the loading control of miR-296, and glyceraldehyde phosphate dehydrogenase (GAPDH) was used as the loading control of AGAP2-AS1 and NOTCH2. Primer sequences were seen in Supplementary Table 1. The 2^−^^ΔΔCt^ method was used for data analysis.

### Western blot analysis

Proteins in tissues and cells were extracted. The loading quantity of the sample was adjusted to 30 µg by deionized water. Proteins were conducted with 10% sodium dodecyl sulfate-polyacrylamide gel electrophoresis and transferred onto membranes, which were blocked with 4% skim milk powder at 4 °C overnight and incubated with primary antibodies NOTCH2 (1:1000) and GAPDH (1:2000, both from Abcam) for 1 h. Afterward, the membranes were incubated with relative secondary antibodies (Abcam) for 1 h, soaked in enhanced chemiluminescent reagent (Pierce Manufacturing Inc., WI, USA) for 1 min, and covered with plastic wrap, then observed through X-ray. GAPDH was taken as the internal reference and the relative protein expression was calculated.

### Subcutaneous tumorigenesis in nude mice

Balb/C nude mice aged 4 w that acquired from Hunan SJA Laboratory Animal Co., Ltd. (Hunan, China) were fed under a specific pathogen-free environment at 26–28 °C, with humidity of 40–60%, 12 h day/night cycle and sterile water and food.

The nude mice were subcutaneously injected with 0.1 mL lung cancer cells (2 × 10^6^/per mouse) and the grouping was in line with cell grouping (*n* = 6). The spirit, diet, defecation, and activity of the mice were observed each day. From the 5th day of the injection, the length diameter (a) and width diameter (b) was measured by a caliper every 5 days. Tumor volume = 0.5 × *a* × *b*^2^. The tumor growth was observed and the mice were euthanized by carbon dioxide on the 30th day. The xenografts were weighed and observed.

### Statistical analysis

All data analyses were conducted using SPSS 21.0 software (IBM Corp. Armonk, NY, USA). The measurement data were expressed as mean ± standard deviation. The unpaired *t* test was performed for comparisons between two groups and one-way analysis of variance (ANOVA) was used for comparisons among multiple groups, and Tukey’s post hoc test was used for pairwise comparisons after one-way ANOVA. *P* value < 0.05 was indicative of a statistically significant difference.

## Supplementary information

supplementary figure 1

supplementary figure 2

supplementary figure 3

supplementary figure 4

supplementary figure 5

suppl6ntary figure 6

supplementary figure 7

supplementary figure 8

supplementary figure legends

supplementary tables
