# Supplementary figures and images for: M2 macrophage-derived exosomal long non-coding RNA AGAP2-AS1 enhances radiotherapy immunity in lung cancer by reducing microRNA-296 and elevating NOTCH2
Source: Cell Death Dis. 2021 May 10;12(5):467. doi: 10.1038/s41419-021-03700-0 (PMC8110970; doi:10.1038/s41419-021-03700-0)

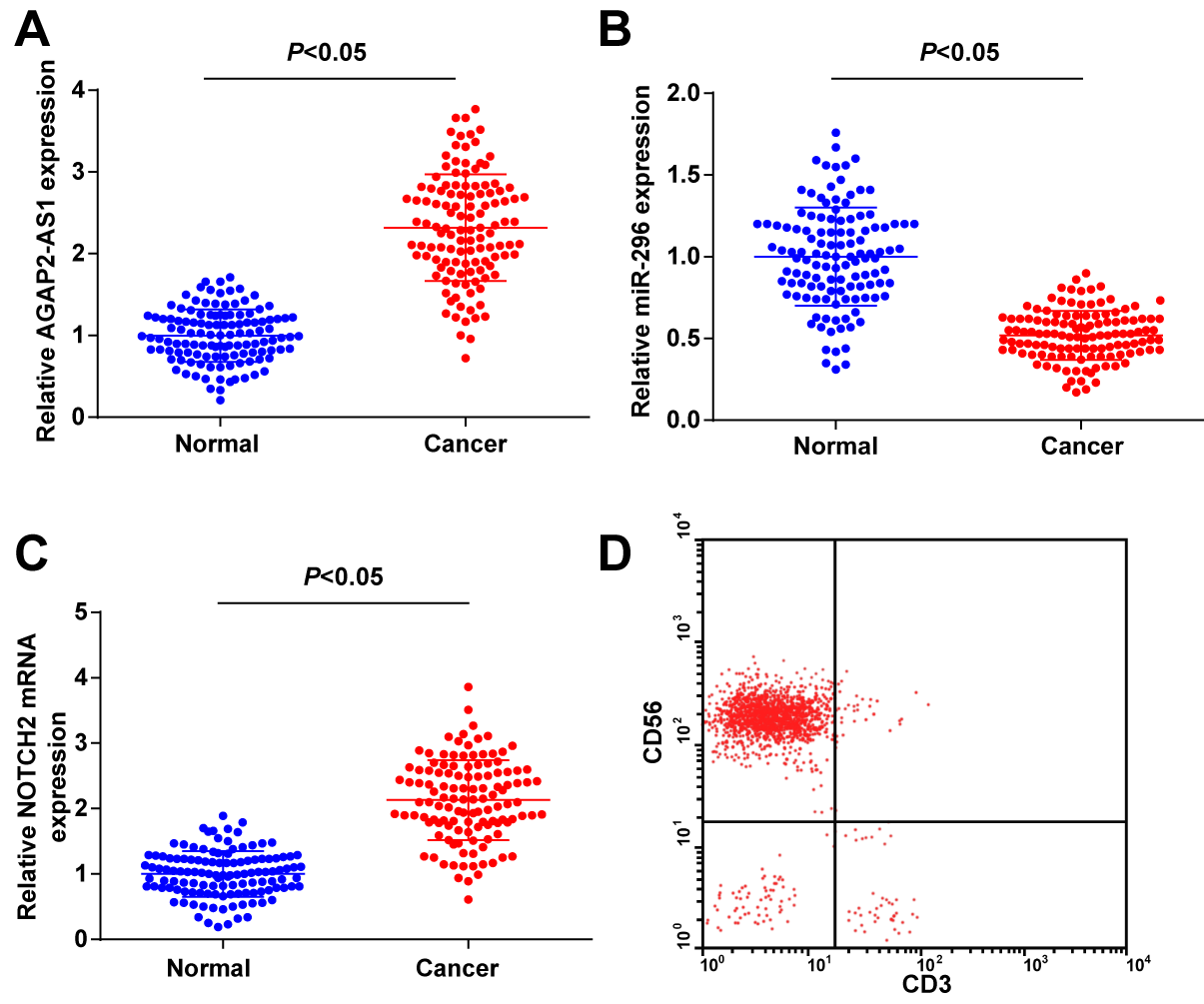

Supplement: Supplementary file 1 — supplementary figure 1 [file 41419_2021_3700_MOESM1_ESM.tif]

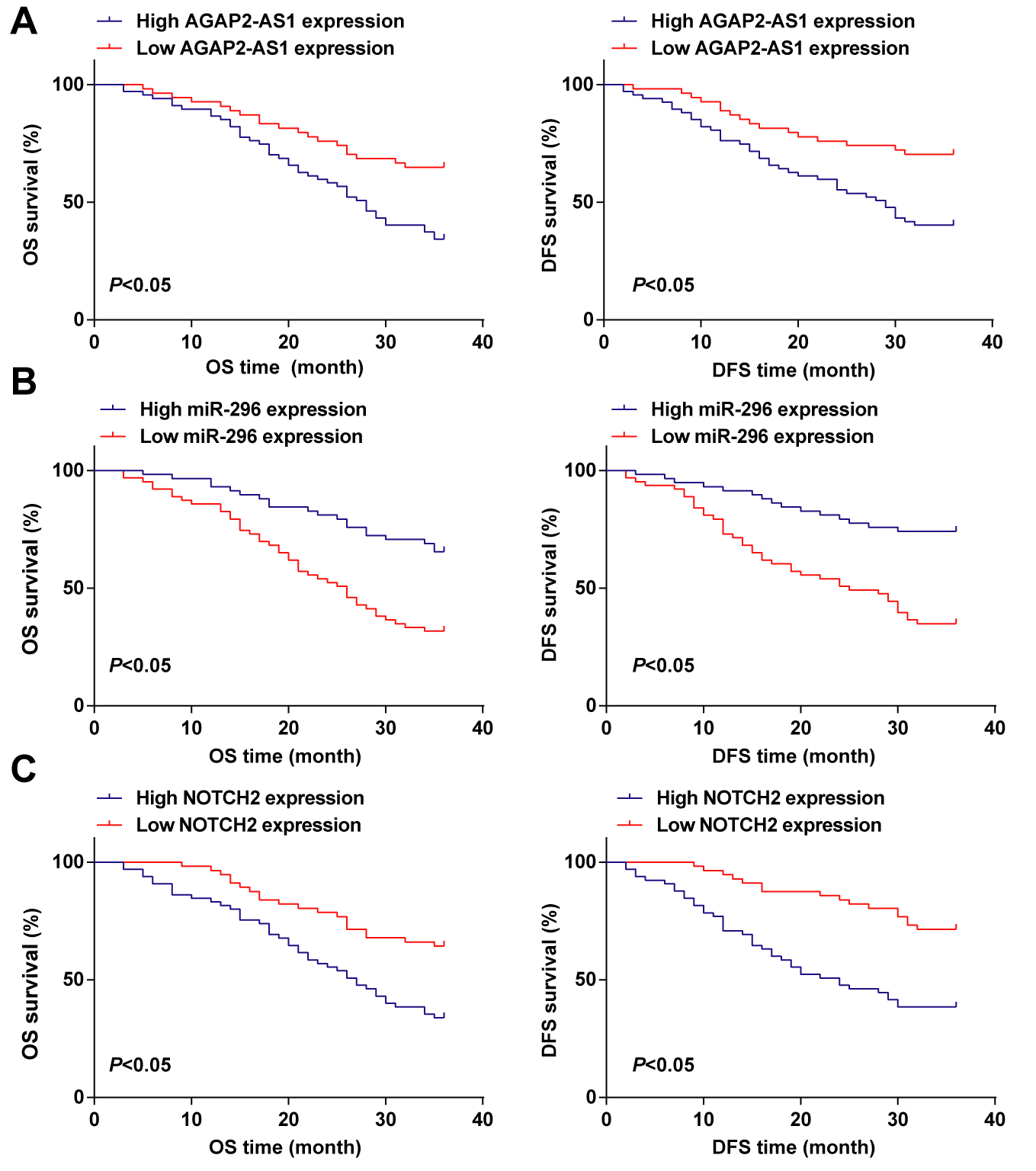

Supplement: Supplementary file 2 — supplementary figure 2 [file 41419_2021_3700_MOESM2_ESM.tif]

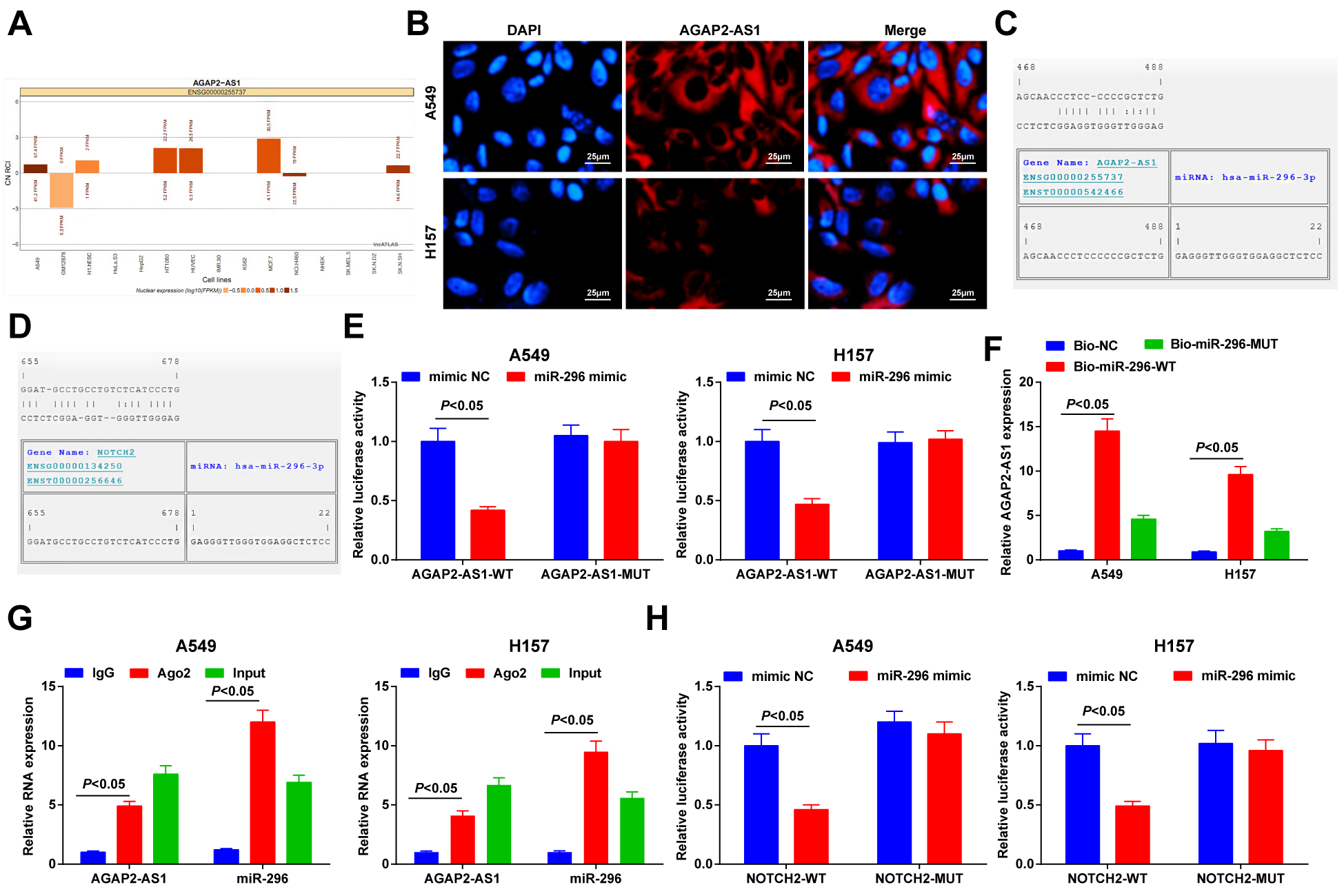

Supplement: Supplementary file 3 — supplementary figure 3 [file 41419_2021_3700_MOESM3_ESM.tif]

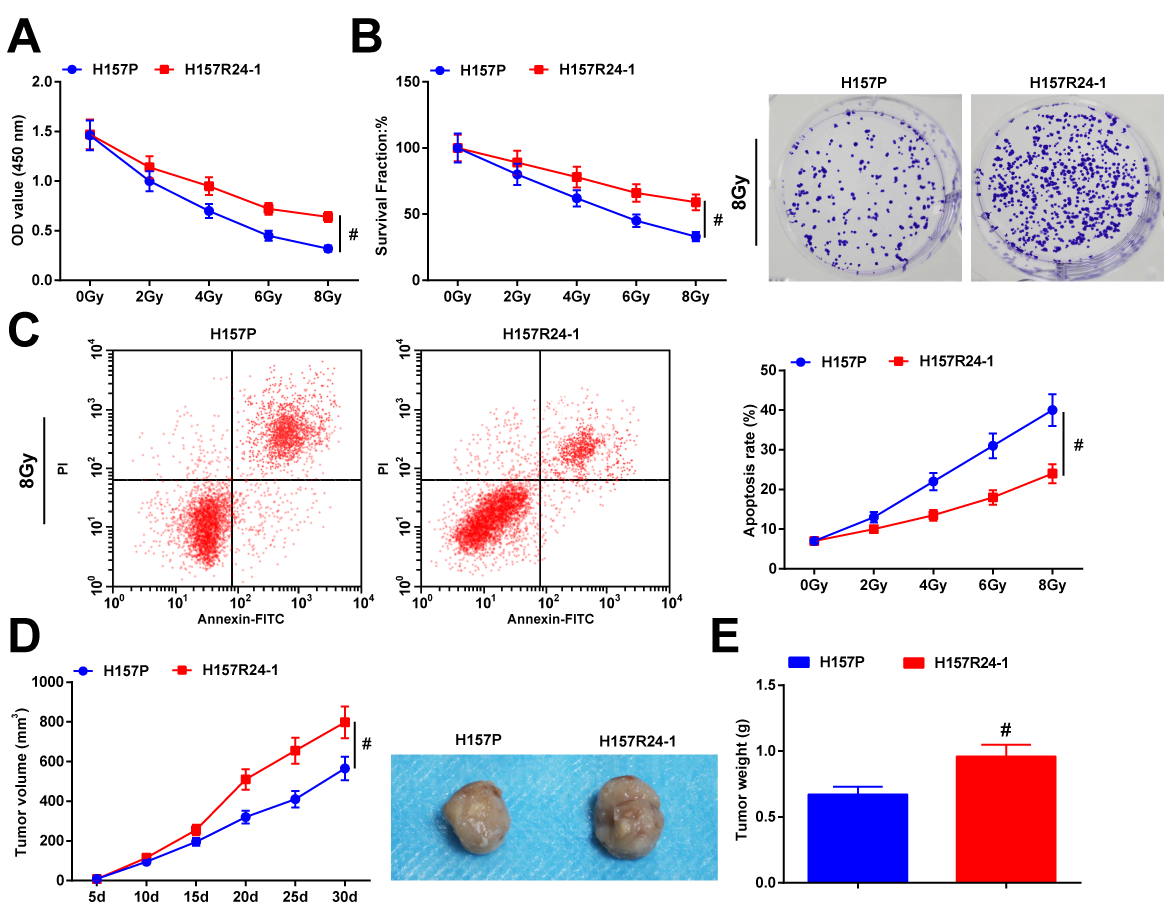

Supplement: Supplementary file 4 — supplementary figure 4 [file 41419_2021_3700_MOESM4_ESM.tif]

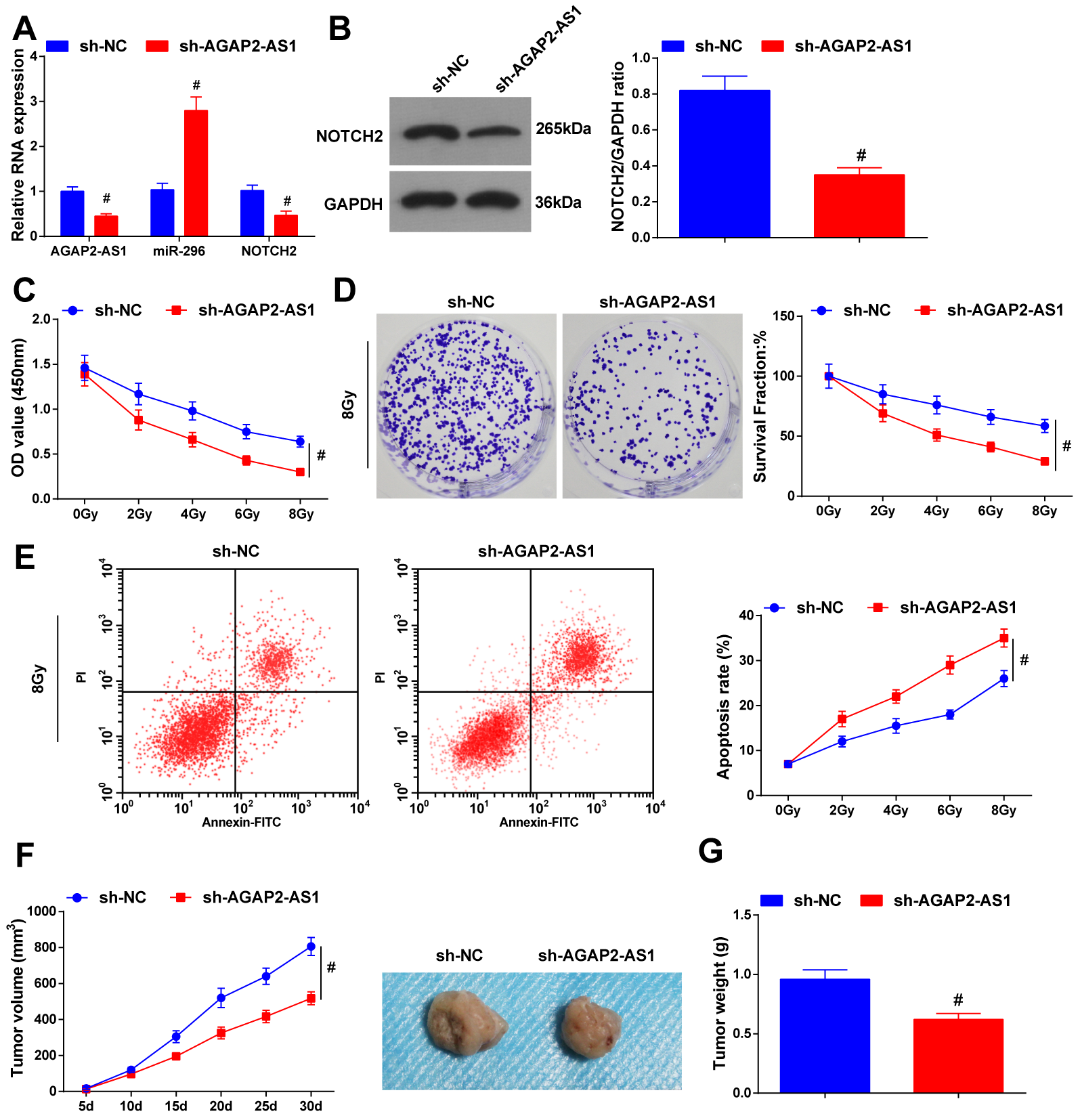

Supplement: Supplementary file 5 — supplementary figure 5 [file 41419_2021_3700_MOESM5_ESM.tif]

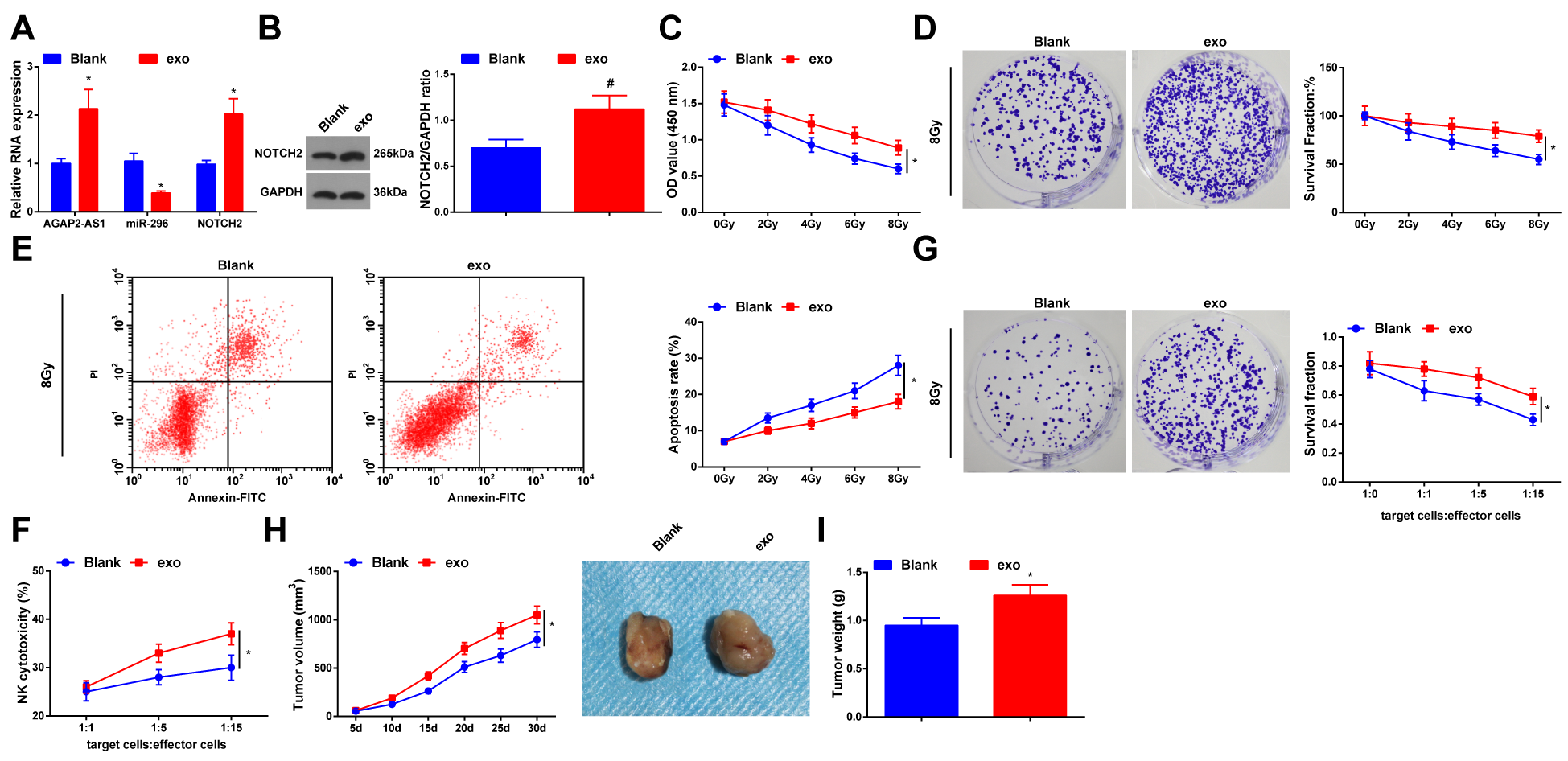

Supplement: Supplementary file 6 — suppl6ntary figure 6 [file 41419_2021_3700_MOESM6_ESM.tif]

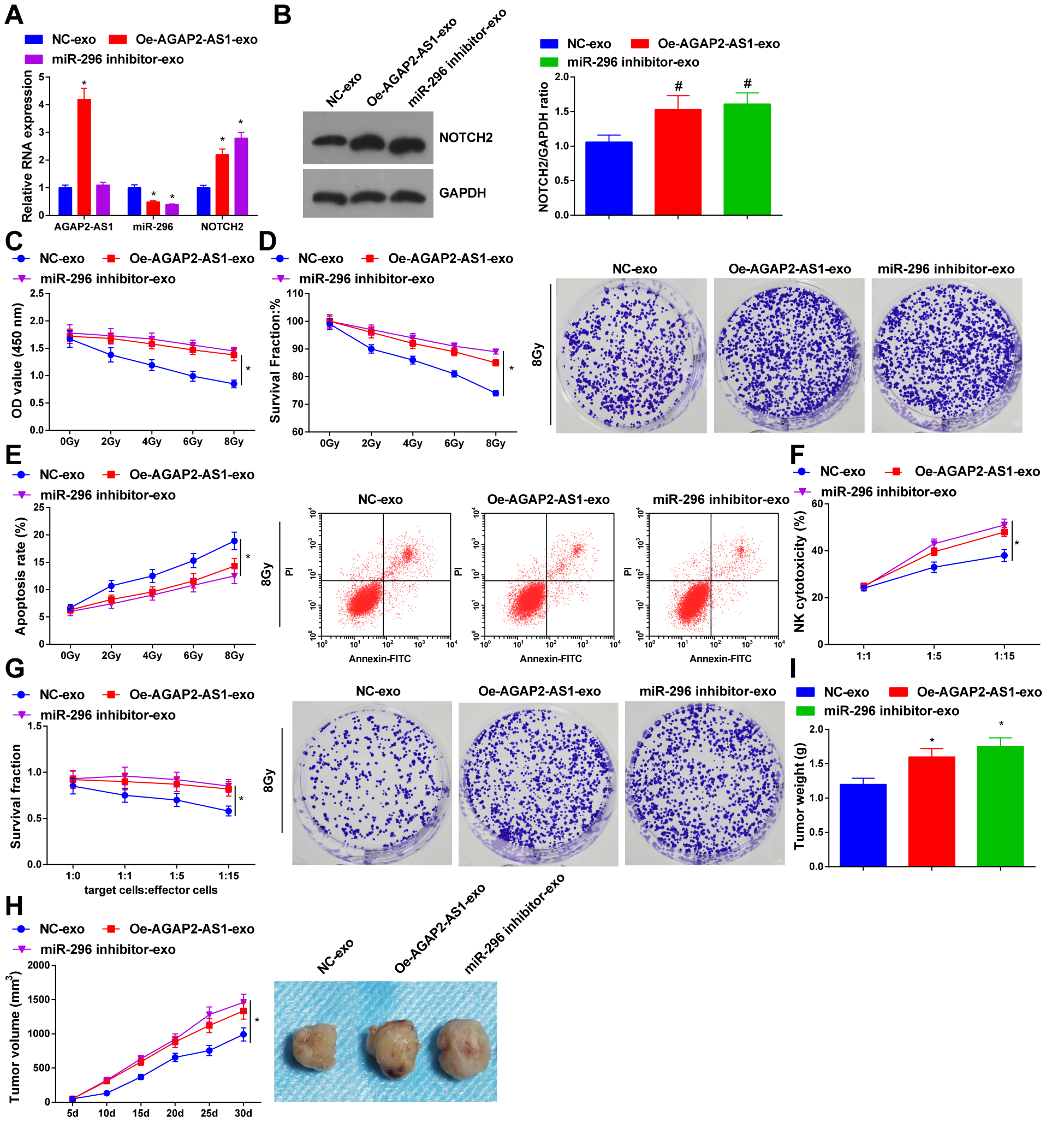

Supplement: Supplementary file 7 — supplementary figure 7 [file 41419_2021_3700_MOESM7_ESM.tif]

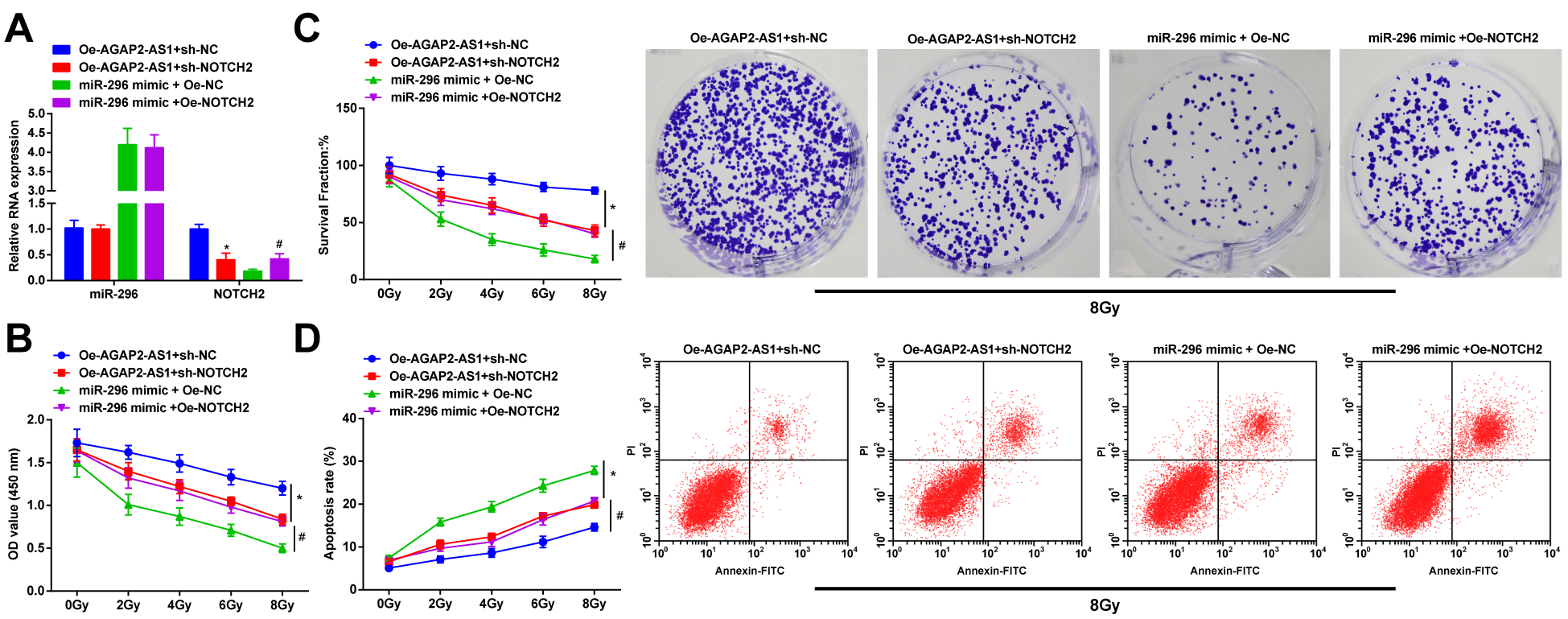

Supplement: Supplementary file 8 — supplementary figure 8 [file 41419_2021_3700_MOESM8_ESM.tif]
