# Supplementary material for: M2 macrophage-derived exosomal long non-coding RNA AGAP2-AS1 enhances radiotherapy immunity in lung cancer by reducing microRNA-296 and elevating NOTCH2
Source: Cell Death Dis. 2021 May 10;12(5):467. doi: 10.1038/s41419-021-03700-0 (PMC8110970; doi:10.1038/s41419-021-03700-0)
Supplement: Supplementary file 9 — supplementary figure legends [file 41419_2021_3700_MOESM9_ESM.docx]

**Supplementary Figure 1** AGAP2-AS1 and NOTCH2 are overexpressed while miR-296 is lowly expressed in lung cancer tissues. A, AGAP2-AS1 expression in lung cancer tissues was detected by RT-qPCR; B, miR-296 expression in lung cancer tissues was detected by RT-qPCR; C, NOTCH2 expression in lung cancer tissues was detected by RT-qPCR. D, The purity of NK cells was detected by flow cytometry was (90.6 ± 6.2)%. The measurement data were expressed as mean ± standard deviation, and analyzed by t test.

**Supplementary Figure 2** AGAP2-AS1 plays a predictive role in prognosis of lung cancer patients. A, The relationship between AGAP2-AS1 expression and OS and DFS; B, The relationship between miR-296 expression and OS and DFS; C, The relationship between NOTCH2 expression and OS and DFS.

**Supplementary Figure 3** AGAP2-AS1 negatively regulates miR-296 and NOTCH2 is a target gene of miR-296. A, subcellular localization of AGAP2-AS1 was analyzed by an online analysis website; B, subcellular localization of AGAP2-AS1 was analyzed by FISH assay; C, target relation between AGAP2-AS1 and miR-296 was predicted by an online analysis website; D, target relation between miR-296 and NOTCH2 was predicted by an online analysis website; E, regulatory relation between AGAP2-AS1 and miR-296 was confirmed by dual luciferase report gene assay; F, binding relation between AGAP2-AS1 and miR-296 clarified by RNA pull-down assay; G, binding of miR-296 and AGAP2-AS1 was assessed by RIP assay; H, regulatory relation between miR-296 and NOTCH2 was confirmed by dual luciferase report gene assay; N = 3; the measurement data were expressed as mean ± standard deviation, unpaired t-test was performed for comparisons between two groups, comparison among two-factor multiple groups were analyzed by two-way ANOVA and Tukey’s post hoc test was used for pairwise comparisons after ANOVA.

**Supplementary Figure 4** Radioresistant lung cancer cells have stronger radioresistance ability than parent cells. A, viability of radioresistant lung cancer cells and parental cells was determined by CCK-8 assay; B, colony formation ability of radioresistant lung cancer cells H157R24-1 and parental cells H157P was detected by colony formation assay; C, apoptosis of radioresistant lung cancer cells H157R24-1 and parent cells H157P was detected by flow cytometry; D, volume of xenografts from nude mice that had been injected with radioresistant lung cancer cells H157R24-1 and parental cells H157P; E, weight of xenografts from nude mice that had been injected with radioresistant lung cancer cells H157R24-1 and parental cells H157P; N = 3 in cellular experiment and n = 6 in animal experiment;# *P* < 0.05 *vs* H157P cells; the measurement data were expressed as mean ± standard deviation, one-way ANOVA was used for comparisons among multiple groups and Tukey’s post hoc test was used for pairwise comparisons after one-way ANOVA.

**Supplementary Figure 5** Inhibited AGAP2-AS1 restricts radioresistance of radioresistant lung cancer cells. A, expression of AGAP2-AS1, miR-296 and NOTCH2 in H157R24-1 cells was detected by RT-qPCR; B, protein expression of NOTCH2 in H157R24-1 cells was determined by Western blot analysis; C, viability of radioresistant lung cancer cells H157R24-1 was measured using CCK-8 assay; D, colony formation ability of radioresistant lung cancer cells H157R24-1 was evaluated by colony formation assay; E, apoptosis of radioresistant lung cancer cells H157R24-1 was determined by flow cytometry; F, volume of xenografts from nude mice that had been injected with radioresistant lung cancer cells H157R24-1; G, weight of xenografts from nude mice that had been injected with radioresistant lung cancer cells H157R24-1; N = 3 in cellular experiment and n = 6 in animal experiment; # *P* < 0.05 *vs* the sh-NC group ; the measurement data were expressed as mean ± standard deviation, one-way ANOVA was used for comparisons among multiple groups and Tukey’s post hoc test was used for pairwise comparisons after one-way ANOVA.

**Supplementary Figure 6** Macrophage-derived exosomes strengthen radioresistance of radioresistant lung cancer cells. A, expression of AGAP2-AS1, miR-296 and NOTCH2 in H157R24-1 cells was detected by RT-qPCR; B, protein expression of NOTCH2 in H157R24-1 cells was determined by Western blot analysis; C, viability of radioresistant lung cancer cells H157R24-1 was measured using CCK-8 assay; D, colony formation ability of radioresistant lung cancer cells H157R24-1 was evaluated by colony formation assay; E, apoptosis of radioresistant lung cancer cells H157R24-1 was determined by flow cytometry; F, NK cells-mediated cytotoxicity against radioresistant lung cancer cells H157R24-1 was detected by LDH cytotoxic assay; G, NK cells-mediated cytotoxicity against radioresistant lung cancer cells H157R24-1 was detected by colony formation assay; H, volume of xenografts from nude mice that had been injected with radioresistant lung cancer cells H157R24-1; I, weight of xenografts from nude mice that had been injected with radioresistant lung cancer cells H157R24-1; N = 3 in cellular experiment and n = 6 in animal experiment; # *P* < 0.05 *vs* the blank group in H157R24-1; the measurement data were expressed as mean ± standard deviation, one-way ANOVA was used for comparisons among multiple groups and Tukey’s post hoc test was used for pairwise comparisons after one-way ANOVA.

**Supplementary Figure 7** Macrophage-derived exosomes overexpress AGAP2-AS1 or reduce miR-296 to strengthen radioresistance of radioresistant lung cancer cells. A, expression of AGAP2-AS1, miR-296 and NOTCH2 in H157R24-1 cells was detected by RT-qPCR; B, protein expression of NOTCH2 in H157R24-1 cells was determined by Western blot analysis; C, viability of radioresistant lung cancer cells H157R24-1 was measured using CCK-8 assay; D, colony formation ability of radioresistant lung cancer cells H157R24-1 was evaluated by colony formation assay; E, apoptosis of radioresistant lung cancer cells H157R24-1 was determined by flow cytometry; F, NK cells-mediated cytotoxicity against radioresistant lung cancer cells H157R24-1 was detected by LDH cytotoxic assay; G, NK cells-mediated cytotoxicity against radioresistant lung cancer cells H157R24-1 was detected by colony formation assay; H, volume of xenografts from nude mice that had been injected with radioresistant lung cancer cells H157R24-1; I, weight of xenografts from nude mice that had been injected with radioresistant lung cancer cells H157R24-1; N = 3 in cellular experiment and n = 6 in animal experiment; * *P* < 0.05 *vs* the NC-exo group; the measurement data were expressed as mean ± standard deviation, one-way ANOVA was used for comparisons among multiple groups and Tukey’s post hoc test was used for pairwise comparisons after one-way ANOVA.

**Supplementary Figure 8** NOTCH2 overexpression rescues the effects of miR-296 up-regulation on radioresistance of radioresistant lung cancer cells. A, detection of transfection efficiency in H157R24-1 cells; B, H157R24-1 cell proliferation was detected by CCK-8 assay; C, H157R24-1 cell survival rate was detected by colony formation assay; D, H157R24-1 cell apoptosis rate was detected by flow cytometry.
