# Supplementary material for: M2 macrophage-derived exosomal long non-coding RNA AGAP2-AS1 enhances radiotherapy immunity in lung cancer by reducing microRNA-296 and elevating NOTCH2
Source: Cell Death Dis. 2021 May 10;12(5):467. doi: 10.1038/s41419-021-03700-0 (PMC8110970; doi:10.1038/s41419-021-03700-0)
Supplement: Supplementary file 10 — supplementary tables [file 41419_2021_3700_MOESM10_ESM.docx]

**Supplementary Table 1** Primer sequence

| Gene | Sequence |
| --- | --- |
| AGAP2-AS1 | F: 5’-TACCTTGACCTTGCTGCTCTC-3’ |
|  | R: 5’-TGTCCCTTAATGACCCCATCC-3’ |
| miR-296 | F: 5’-TGCCTAATTCAGAGGGTTGG-3’ |
|  | R: 5’-AACGCTTCACGAATTTGCGT-3’ |
| U6 | F: 5’-CGCTTCGGCAGCACATATAC-3’ |
|  | R: 5’-TTCACGAATTTGCGTGTCAT-3’ |
| NOTCH2 | F: 5’-CAACCGCCAGTGTGTTCAAG-3’ |
|  | R: 5’-GAGCCATGCTTACGCTTTCG-3’ |
| GAPDH | F: 5’-GGGAGCCAAAAGGGTCAT-3’ |
|  | R: 5’-GAGTCCTTCCACGATACCAA-3’ |

Note: F, forward; R, reverse; AGAP2-AS1, AGAP2 antisense RNA 1; miR-296, microRNA-296; NOTCH2, notch homolog protein 2; GAPDH, glyceraldehyde phosphate dehydrogenase.

**Supplementary Table 2** Relation between expression levels of AGAP2-AS1, miR-296 and NOTCH2 with clinicopathological characteristics of lung cancer patients [n (%)]

| Clinicopathological characteristics | n | AGAP2-AS1 expression | | *P* |
| --- | --- | --- | --- | --- |
|  |  | High expression  (n = 67) | Low expression（n = 54） |  |
| Age (year) |  |  |  | 0.508 |
| < 53 | 52 | 27 | 25 |  |
| ≥ 53 | 69 | 40 | 29 |  |
| Gender |  |  |  | 0.366 |
| male | 73 | 38 | 35 |  |
| female | 48 | 29 | 19 |  |
| Pathological pattern |  |  |  | 0.088 |
| squamous carcinoma | 64 | 31 | 33 |  |
| adenocarcinoma | 57 | 37 | 21 |  |
| Tumor differentiation |  |  |  | 0.153 |
| high | 41 | 19 | 22 |  |
| mild + low | 80 | 48 | 32 |  |
| TNM |  |  |  | 0.006 |
| I + II | 59 | 25 | 34 |  |
| III + IV | 62 | 42 | 20 |  |
| LNM |  |  |  | 0.030 |
| no | 56 | 25 | 31 |  |
| yes | 65 | 42 | 23 |  |

| Clinicopathological characteristics | n | miR-296 expression | | *P* |
| --- | --- | --- | --- | --- |
|  |  | High expression  (n = 58) | Low expression（n = 63） |  |
| Age (year) |  |  |  | 0.854 |
| < 53 | 52 | 24 | 28 |  |
| ≥ 53 | 69 | 34 | 35 |  |
| Gender |  |  |  | 0.577 |
| male | 73 | 33 | 40 |  |
| female | 48 | 25 | 23 |  |
| Pathological pattern |  |  |  | 0.716 |
| squamous carcinoma | 64 | 32 | 32 |  |
| adenocarcinoma | 57 | 26 | 31 |  |
| Tumor differentiation |  |  |  | 0.701 |
| high | 41 | 21 | 20 |  |
| mild + low | 80 | 37 | 43 |  |
| TNM |  |  |  | 0.004 |
| I + II | 59 | 20 | 39 |  |
| III + IV | 62 | 38 | 24 |  |
| LNM |  |  |  | 0.018 |
| no | 56 | 20 | 36 |  |
| yes | 65 | 38 | 27 |  |

| Clinicopathological characteristics | n | NOTCH2 expression | | *P* |
| --- | --- | --- | --- | --- |
|  |  | High expression  (n = 65) | Low expression（n = 56） |  |
| Age (year) |  |  |  | 0.581 |
| < 53 | 52 | 26 | 26 |  |
| ≥ 53 | 69 | 39 | 30 |  |
| Gender |  |  |  | 0.578 |
| male | 73 | 41 | 32 |  |
| female | 48 | 24 | 24 |  |
| Pathological pattern |  |  |  | 0.466 |
| squamous carcinoma | 64 | 32 | 32 |  |
| adenocarcinoma | 57 | 33 | 24 |  |
| Tumor differentiation |  |  |  | 0.255 |
| high | 41 | 19 | 22 |  |
| mild + low | 80 | 46 | 34 |  |
| TNM |  |  |  | 0.029 |
| I + II | 59 | 38 | 21 |  |
| III + IV | 62 | 27 | 35 |  |
| LNM |  |  |  | 0.018 |
| no | 56 | 37 | 19 |  |
| yes | 65 | 28 | 37 |  |

Note: AGAP2-AS1, AGAP2 antisense RNA 1; miR-296, microRNA-296; NOTCH2, notch homolog protein 2; TNM, tumor, node and metastasis; LNM, lymph node metastasis. Data in this table are enumeration data and analyzed by chi-square test.
